# The Quality of Methods Reporting in Parasitology Experiments

**DOI:** 10.1371/journal.pone.0101131

**Published:** 2014-07-30

**Authors:** Oscar Flórez-Vargas, Michael Bramhall, Harry Noyes, Sheena Cruickshank, Robert Stevens, Andy Brass

**Affiliations:** 1 Bio-health Informatics Group, School of Computer Science, University of Manchester, Manchester, United Kingdom; 2 School of Biological Science, University of Liverpool, Liverpool, United Kingdom; 3 Manchester Immunology Group, Faculty of Life Science, University of Manchester, Manchester, United Kingdom; INSERM U1094, University of Limoges School of Medicine, France

## Abstract

There is a growing concern both inside and outside the scientific community over the lack of reproducibility of experiments. The depth and detail of reported methods are critical to the reproducibility of findings, but also for making it possible to compare and integrate data from different studies. In this study, we evaluated in detail the methods reporting in a comprehensive set of trypanosomiasis experiments that should enable valid reproduction, integration and comparison of research findings. We evaluated a subset of other parasitic (*Leishmania*, *Toxoplasma*, *Plasmodium*, *Trichuris* and *Schistosoma*) and non-parasitic (*Mycobacterium*) experimental infections in order to compare the quality of method reporting more generally. A systematic review using PubMed (2000–2012) of all publications describing gene expression in cells and animals infected with *Trypanosoma spp* was undertaken based on PRISMA guidelines; 23 papers were identified and included. We defined a checklist of essential parameters that should be reported and have scored the number of those parameters that are reported for each publication. Bibliometric parameters (impact factor, citations and h-index) were used to look for association between Journal and Author status and the quality of method reporting. Trichuriasis experiments achieved the highest scores and included the only paper to score 100% in all criteria. The mean of scores achieved by *Trypanosoma* articles through the checklist was 65.5% (range 32–90%). Bibliometric parameters were not correlated with the quality of method reporting (Spearman's rank correlation coefficient <−0.5; *p*>0.05). Our results indicate that the quality of methods reporting in experimental parasitology is a cause for concern and it has not improved over time, despite there being evidence that most of the assessed parameters do influence the results. We propose that our set of parameters be used as guidelines to improve the quality of the reporting of experimental infection models as a pre-requisite for integrating and comparing sets of data.

## Introduction

In this study, we evaluated the reported information on experimental methods in published infectious disease experiments that should enable a valid comparison of research findings. It has been claimed that most published research findings are false [1] and concern about this is spreading beyond the scientific community, making the cover of The Economist recently [2], and potentially undermining public trust in science. Amongst the scientific community there is a growing concern over the related problem of lack of reproducibility [3], [4]. The depth and detail of reported methods directly contributes to the replicability, reproducibility and comparability of experimental work. Replicability is the exact repetition of an experiment to obtain the same results, reproducibility is the repetition of an experiment with small modifications, e.g. the changes that will inevitably occur when conducting the same experiment in different laboratories [5], [6]. If results are replicable but not reproducible they may be of little value since they are likely to be idiosyncratic to the precise conditions used and further inference from the results will be problematic. Comparability is essential to facilitate translational discoveries by making it possible to aggregate data from multiple experiments in a single meta-analysis and answering questions not addressed by the original investigators. The information reported in the Materials & Methods section of an article plays a fundamental role in achieving this aim. In the biomedical field, for instance, the Uniform Guidelines of the International Committee of Medical Journal Editors state that authors should include technical information in sufficient detail to allow the experiment to be repeated by other workers [7]. However, the guidelines are not strictly adhered to and, consequently, the lack of methodological information can make the tasks of replicating, reproducing or comparing results by non-specialists in a field problematic.

Over the past decade sets of minimum items of information have been published that should be reported about a dataset or an experimental process [8]. This allows readers not only to unambiguously interpret and critically evaluate the conclusions reached, but also to potentially replicate, reproduce and compare the experiments. The minimum information checklist or guidelines seek to promote transparency in experimental reporting, enhance accessibility to data and support effective quality assessment, which increases the general value of data, and therefore of the scientific evidence. In this sense, some standard initiatives, such as the Minimum Information About a Microarray Experiment (MIAME) [9] and the Minimum Information About a Proteomics Experiment (MIAPE) [10], have been adopted by several journals, such as Nature Genetics or the Journal of Proteomics, as a requirement for publication.

To address the issue of reproducibility in the context of biomedical experiments, we looked at experimental infection models with a particular focus on the trypanosomiases, which are a widespread group of complex infectious diseases caused by flagellated protozoa of the genus *Trypanosoma*. These infections affect humans and animals, often with fatal consequences unless treated. In humans, African (sleeping sickness) and American (Chagas disease) trypanosomiases are responsible for considerable morbidity and mortality, affecting millions of people every year [11]–[13]. Moreover, human economic welfare in Africa is also affected by these diseases due to loss of livestock production [14]. The outcome of infection with both American and African trypanosomes depends on both the host and parasite genetic background as well as on environmental variation [15]–[17]. In addition, the trypanosomiases have been labelled as “neglected” because their study hovers in the margins of international health; there is a smaller investment in their research and development and as a result they are less well understood. Hence, an important task is to integrate and compare data from their studies in order to augment the value of this data.

Many studies have been carried out to explore the physiopathology of sleeping sickness and Chagas disease, as well as their genetics. At the time of writing, a PubMed search from 2000–2013 retrieved 1558 and 4248 journal articles containing the MeSH (Medical Subject Headings) terms “Trypanosomiasis, African” and “Chagas disease”, respectively. Despite the large amount of published research, our understanding of the underlying mechanisms involved in these diseases is still limited. It is likely that this can be partly explained by the inherent difficulty in making direct comparisons between the results of independent *Trypanosoma* infection experiments.

Currently we have data from studies carried out in experimental models of trypanosomiasis. However, a considerable part of this evidence is controversial or contradictory; probably stemming from differences in pre-analytical, analytical and post-analytical variables, as well as experimental design and data analysis. In Chagas diseases, for instance, the role played by the Th17 immune response, T regulatory cells and Nitric Oxide may be critical to the outcome of infection [18]–[20] or these immune factors may have opposing effects or not be required [21]–[23]. Therefore, it is important to know how the data were produced in order to deal not only with the biological complexity of these diseases, but also to permit the replicability, reproducibility and, especially in the case of contradictory results, the comparability of research findings. In order to assess how easy it would be to replicate, reproduce or compare experiments we have undertaken a systematic review of all publications describing gene expression experiments in model organisms infected with these parasites. We have defined a list of essential parameters describing the parasite, the host and the infection that should be reported and for each experiment we have scored the number of those parameters that are reported. In order to determine whether our findings can be generalised to other diseases we have used the same method to assess a subset of papers on *Leishmania*, *Toxoplasma* and *Plasmodium*. A subset of papers that utilised the intestinal helminth parasite *Trichuris muris* or *Schistosoma sp*. were used as a comparative control in order to determine the relevance of the checklist in a non-protozoan parasite infection model. In addition, a subset of papers from a non-parasitic infection model (*Mycobacterium*) were used in order to determine whether this issue is unique to parasitology or has wider implications.

## Results

### Search strategy

**Figure 1:**
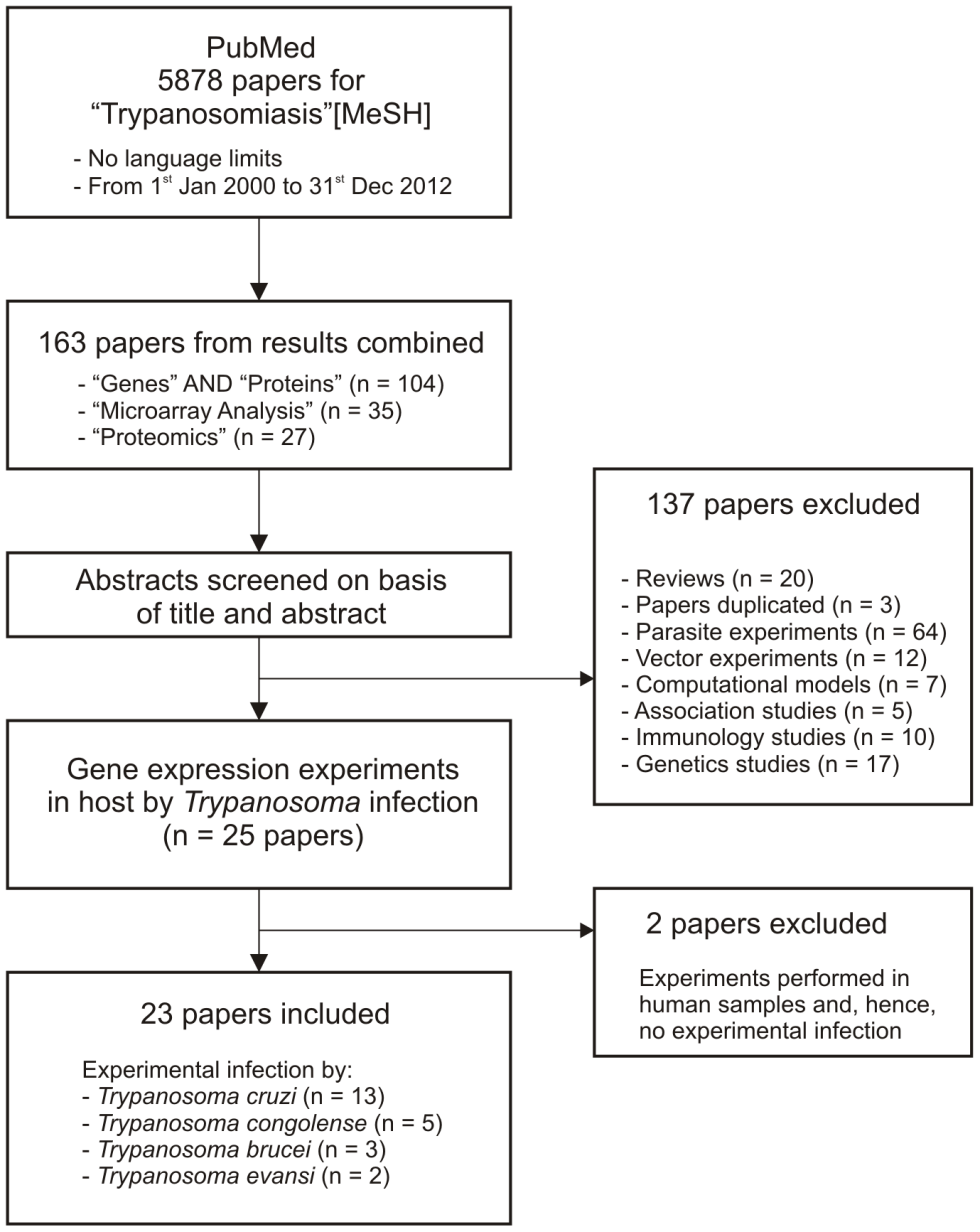
Study selection process for *Trypanosoma* studies.

A total of 23 papers on *Trypanosoma* experiments were identified for inclusion in the review. The search in PubMed provided a total of 5878 references with the MeSH term “Trypanosomiasis”, of which 104 were related with terms “Genes” and “Proteins”, 35 with “Microarray Analysis”, and 27 with “Proteomics”. After adjusting for duplicates 163 remained. The abstracts of these papers were reviewed manually and 139 were discarded because they did not meet the selection criteria (Figure 1 and Table 1). The remaining 23 references [24]–[46] were the corpus of papers identified that reported on gene expression profiling in the host due to an experimental *Trypanosoma* infection. A subset of 10 articles each of the closely related protozoan parasites *Leishmania* [47]–[56], *Toxoplasma* [57]–[66] and *Plasmodium* [67]–[76] were included for comparison. In addition, 10 articles of *Trichuris* [77]–[86] and *Schistosoma* [87]–[96] parasitic worm experiments, and 10 articles of *Mycobacterium* [97]–[106] experiments as a non-parasitic infection model were included in order to contrast the quality of method reporting in *Trypanosoma* experiments to other models and to determine the applicability of the checklist to different experimental systems.

**Table 1:** Studies characteristics in trypanosomiasis: parasite species, experimental infection models and aims of the studies.

Author, year and journal	Parasite	Infection model	Aim
Amin et al., 2010 Am J Trop Med Hyg	*T. b. brucei*	Mouse	Discover genes differentially expressed in brain of mice at the early and late stages of *T. b. brucei* infection.
Chessler et al., 2009 J Immunol	*T. cruzi*	Mouse	Examine the initial host-parasite interaction in vivo by monitoring changes in global host mRNA levels at the site of intradermal infection of mice with *T. cruzi*.
Costales et al., 2009 BMC Genomics	*T. cruzi*	Cell line	Investigate the impact of intracellular *T. cruzi* infection on host cell gene expression.
Garg at al., 2004 Biochem J	*T. cruzi*	Mouse	Characterise the cardiac metabolic response to *T. cruzi* infection and progressive disease severity.
Genovesio et al., 2011 PLoS One	*T. cruzi*	Cell line	Search for human cell factors that play a role during infection by the protozoan parasite *T. cruzi*.
Goldenberg et al., 2009 Microbes Infect	*T. cruzi*	Primary culture (Cardiomyocytes)	Examine gene profiling of *T. cruzi*-infected cardiac myocytes.
Graefe et al., 2006 PLoS One	*T. cruzi*	Mouse	Analyse genome wide expression differences in the spleen at the point at which the immune response diverges between susceptible and resistant mice, and then match the genomic localisation of differential expressed genes with mapped susceptibility loci.
Hashimoto et al., 2005 Int J Parasitol	*T. cruzi*	Cell line	Report the time-course of transcriptional changes in apoptosis-related genes responsive to Fas stimulation in *T. cruzi* infected cells.
Hill et al., 2005 Vet Immunol Immunopathol	*T. congolense*	Cattle	Investigate the transcriptional response of susceptible cattle to trypanosome infection.
Kierstein et al., 2006 Genes Immun	*T. congolense*	Mouse	Explore the ability of more integrated analysis of genetics of trypanotolerance underlying the response to infection and identify pathways involved in trypanotolerance.
Li et al., 2009 Parasitol Res	*T. evansi*	Mouse	Investigate the global gene expression in the liver and spleen of mice after infection with *T. evansi*.
Li et al., 2011 Exp Parasitol	*T. b. brucei*	Mouse	Examine the effects of *T. b. brucei* infection on the liver and spleen of mice at the molecular level.
Lopez et al., 2008 J Immunol	*T. b. rhodesiense*	Mouse, primary culture and cell line	Define the spectrum of host innate immune response genes that are induced during early trypanosome infection in macrophages *ex vivo* as well as macrophages treated *in vitro* with sVSG.
Manque et al., 2011 Infect Immun	*T. cruzi*	Primary culture (Cardiomyocytes)	Characterise the global response of murine cardiomyocytes after infection by trypomastigotes in a carefully controlled progression.
Meade et al., 2009 Mol Immunol	*T. congolense*	Cattle	Determine the expression levels of AMP and APP genes in PBMC isolated from trypanotolerant and trypanosusceptible cattle experimentally infected with *T. congolense*.
Mekata et al., 2012 Parasite Immunol	*T. evansi*	Mouse	Determine what kinds of inflammatory molecules play roles in the pathogenicity of *T. evansi* infection.
Mukherjee et al., 2003 Parasitol Res	*T. cruzi*	Mouse	Identify genes that could contribute to cardiac remodelling as a result of *T. cruzi* infection.
Mukherjee et al., 2008 Genomics	*T. cruzi*	Mouse	Report the patterns of gene expression during the development of murine chagasic heart disease, encompassing several time points in the transition from acute to chronic disease.
Noyes et al., 2009 PLoS One	*T. congolense*	Mouse	Assess the parameters that influence anaemia in murine *T. congolense* infections using mouse strains that differ in their susceptibility to trypanosomiasis.
O'Gorman et al., 2009 BMC Genomics	*T. congolense*	Cattle	Catalogue and analyse gene expression changes in PBMC from trypanotolerant and trypanosusceptible cattle following an experimental challenge with *T. congolense*.
Soares et al., 2010 J Infect Dis	*T. cruzi*	Mouse	Determine alterations in gene expression in the myocardium of mice chronically infected with *T. cruzi*.
Soares et al., 2011 Cell Cycle	*T. cruzi*	Mouse	Evaluate the efficacy of transplantation of BMC to restore the normal transcriptome in the myocardium of mice chronically infected with *T. cruzi*.
Tanowitz et al., 2011 Cell Cycle	*T. cruzi*	Primary culture (Endothelial cells)	Determine the potential molecular mechanisms by which the parasite-derived TXA_2_ modulates Chagas disease progression and limits collateral damage to organs.

### Quality of method reporting

**Figure 2:**
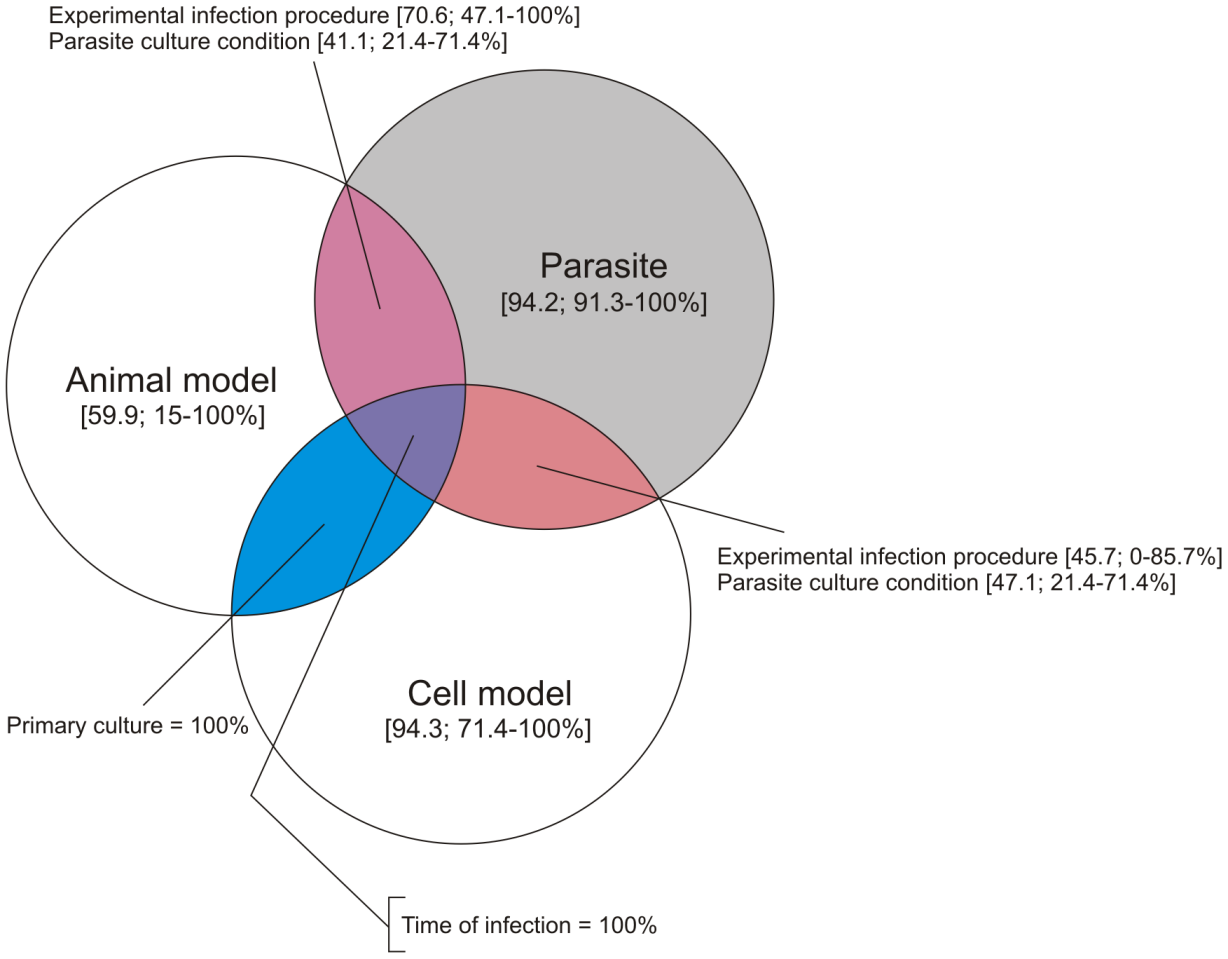
Venn diagram summarising the quality of methods reporting in the three domains of *Trypanosoma* experiments. The average and range of percentages scored of the quality of methods reporting is shown in brackets.

To assess the quality of method reporting in *Trypanosoma* experiments, each paper was checked for reporting of information in three domains: the parasite, the host and the experimental infection. The scores are listed in Tables S1, S2 and S3. A mean of 65.5% (SD  = 15.12%) of the information required to reproduce an experiment was reported in this set of papers. No article met all criteria that should be reported in a *Trypanosoma* experiment according to our checklist (range 32–90%), although two studies [27], [41] scored at 100% out of the available criteria for the parasite and host domains (Tables S1 and S2). The number of articles that met all criteria was higher in the parasite domain (6 out of 23 articles), however the number of criteria met by all the articles was higher in the host domain (7 out of 12 criteria) (Figure 2, Tables S1, S2 and S3). In the experimental infection domain, the inoculum was the only criteria met by all articles, whereas the viability criteria for both cells and parasites were not met in full by any of the studies (Table S3).

### Bibliometric indices

**Figure 3:**
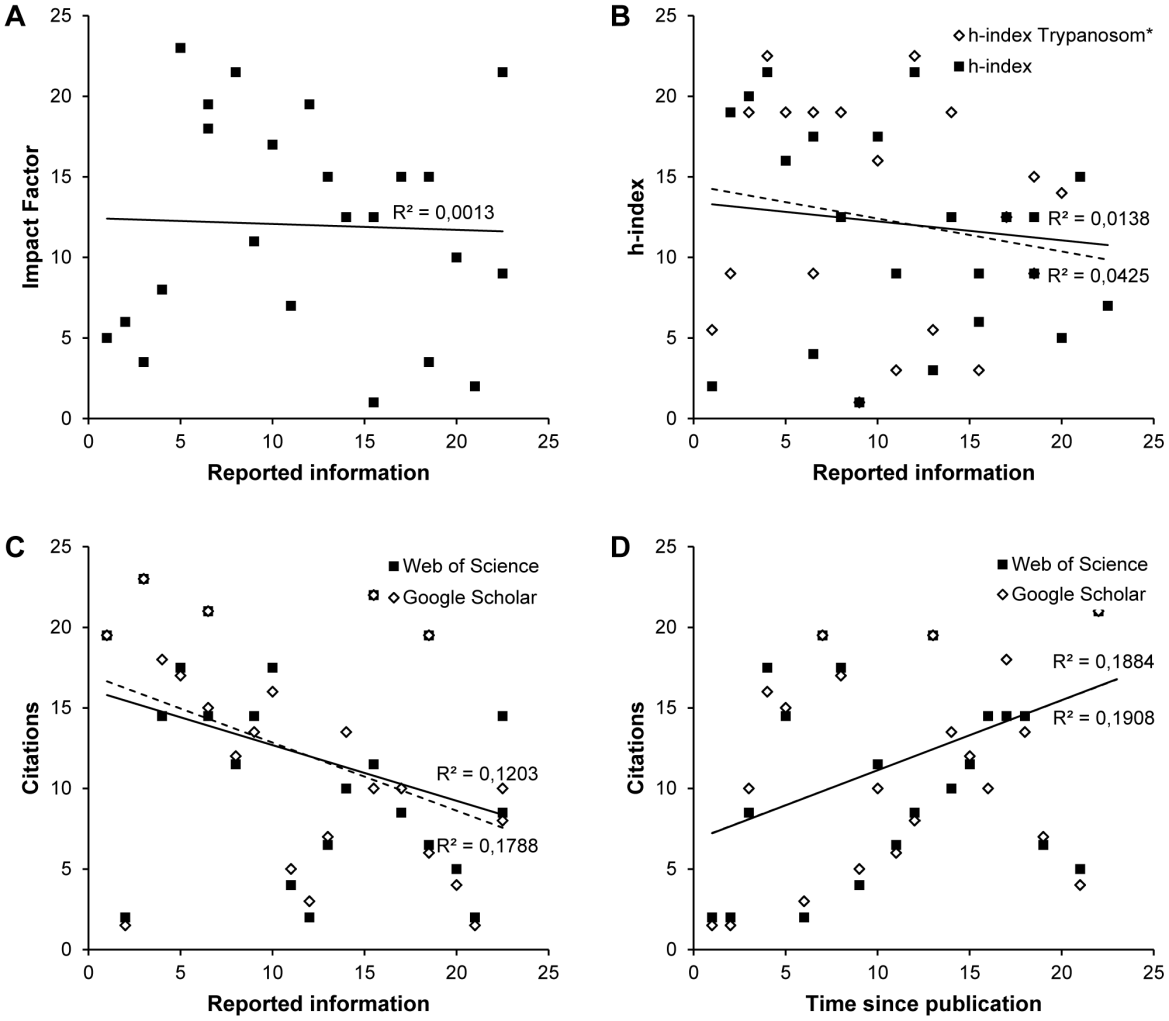
Scatter plots showing the relationship between the quality of methods reporting and the bibliometric indices. Journal impact factor in which the papers were published (A), h-index of the corresponding author (B), and number of citations that the articles have received in other publications (C). Spearman's rank correlation coefficient *r* is shown alongside the regression lines. The figure shows that there is no correlation between the quality of methods reporting and impact factor [*r* = −0.04, *p* = 0.868]. A similar result is shown with h-index, which was searched using the full name of the corresponding author [*r* = −0.12, *p* = 0.593; continuous line] and then filtered by the topic Trypanosom* [*r* = −0.21, *p* = 0.345; broken line]. There is a weak but significant correlation between the quality of methods reporting and the number of citations recorded by Google Scholar [*r* = −0.42, *p* = 0.044; broken line], but not by Web of Science [*r* = −0.35, *p* = 0.105; continuous line]. In order to find out if this association is due to a causal effect of the time of publication, a correlation between the number of citations and the time of publication was done (D), and also a weak but significant correlation was shown with the records of Web of Science [*r* = 0.42, *p* = 0.046; continuous line], but not with Google Scholar [*r* = 0.40, *p* = 0.057; broken line].

**Figure 4:**
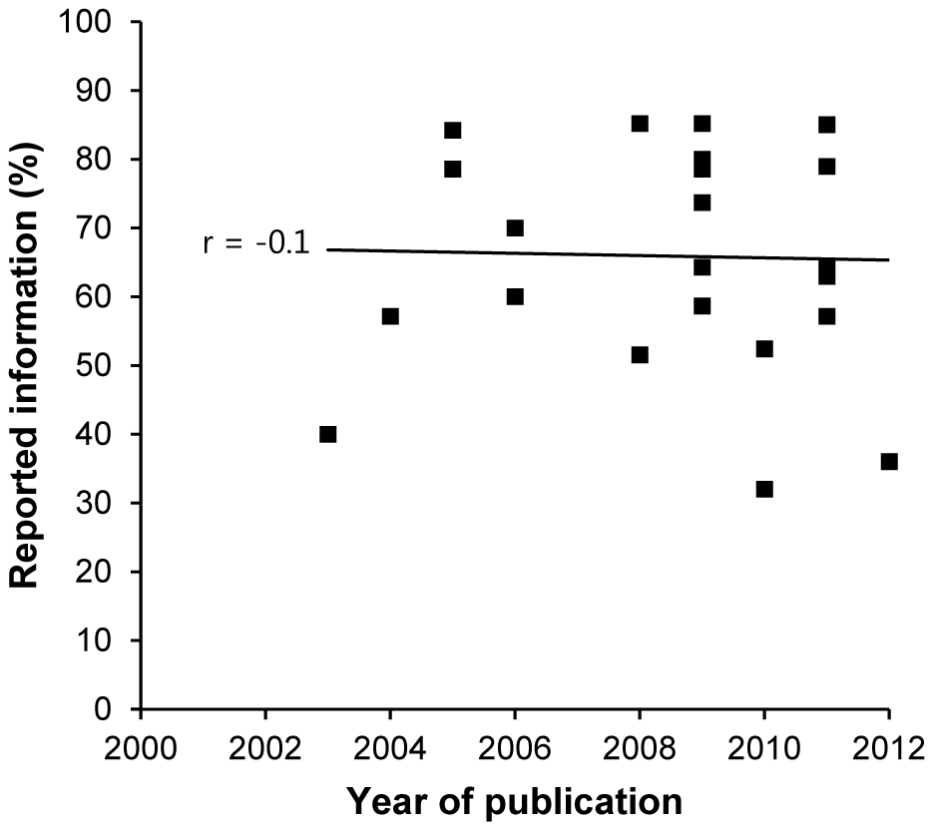
Scatter plots between the reported information in *Trypanosoma* experiments and year of publication. The figure shows that there is no correlation [*p* = 0.711] and that between 2000 and 2012 the quality of methods reporting has remain constant (arithmetic mean  = 65.5%).

Different journals have different criteria for publication in order to enhance the quality of research and to prevent publication of poor findings. However, these safeguards are not always successful; limited space for the method section or forms of bias in the peer review process are some of the issues that have generated serious discussion in several scientific journals [107]. Thus, to discover whether there was an association between bibliometric parameters and the quality of method reporting in *Trypanosoma* experiments, the journal impact factor, the h-index of the corresponding author and the number of citations of the article were compared with the scores for the quality of method reporting. No correlation was observed between method reporting scores and impact factor or h-index (Figures 3A and 3B). However, a significant negative correlation was observed when the scores for method reporting were correlated with the number of citations of the article obtained from Google Scholar (*r* = −0.42; *p* = 0.044, n = 23) but not with citations from the Web of Sciences (*r* = −0.35; *p* = 0.105, n = 23) (Figure 3C). Interpretation of this observation is confounded by the tendency of older papers to have more citations (Google Scholar: *r* = −0.40; *p* = 0.057, n = 23; Web of Sciences: *r* = −0.42; *p* = 0.046, n = 23; Figure 3D). There was no correlation between the quality of method reporting and the year of publication, which remained constant during the last 12 years (Figure 4).

**Figure 5:**
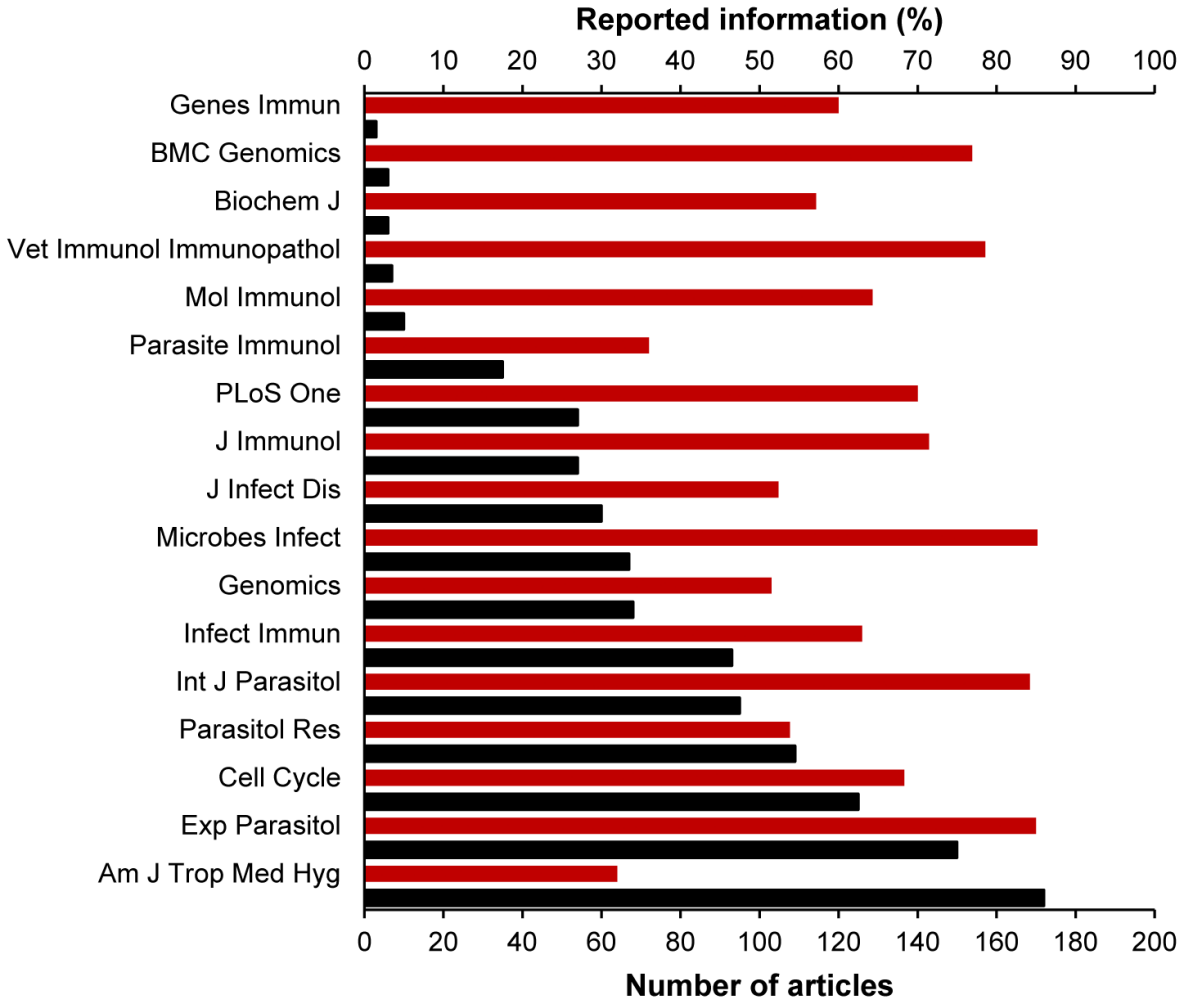
Diagram of articles about Trypanosomiasis[MeSH] published between 2000 and 2012. Number of articles published per journal (black bars) and the percentage of methods reporting (red bars). The figure shows that the quality of method reporting is not related with the number of papers published by any one of the journals.

In order to identify relations between the quality of methods reporting in *Trypanosoma* experiments and the experience of the journal with publishing papers about trypanosomiasis, we compared the scores achieved for the articles (arithmetic mean was calculated for two or more papers) with the number of articles about trypanosomiasis in the journal in which the articles were published. This comparison showed that the number of articles published in any one journal about trypanosomiasis was not associated with an increase in the quality of methods reporting. The journals with most and fewest articles published about trypanosomiasis between 2000 and 2012 were the American Journal of Tropical Medicine and Hygiene with 172 papers and Genes and Immunity with only three papers (Table S4). Nonetheless, the article that received the lowest score in the reported information (32%) was published in the American Journal of Tropical Medicine and Hygiene [40], whereas the mean score for articles published in Genes and Immunity [36] was almost double this value (60%) (Figure 5).

### Comparison with other parasitic diseases

**Figure 6:**
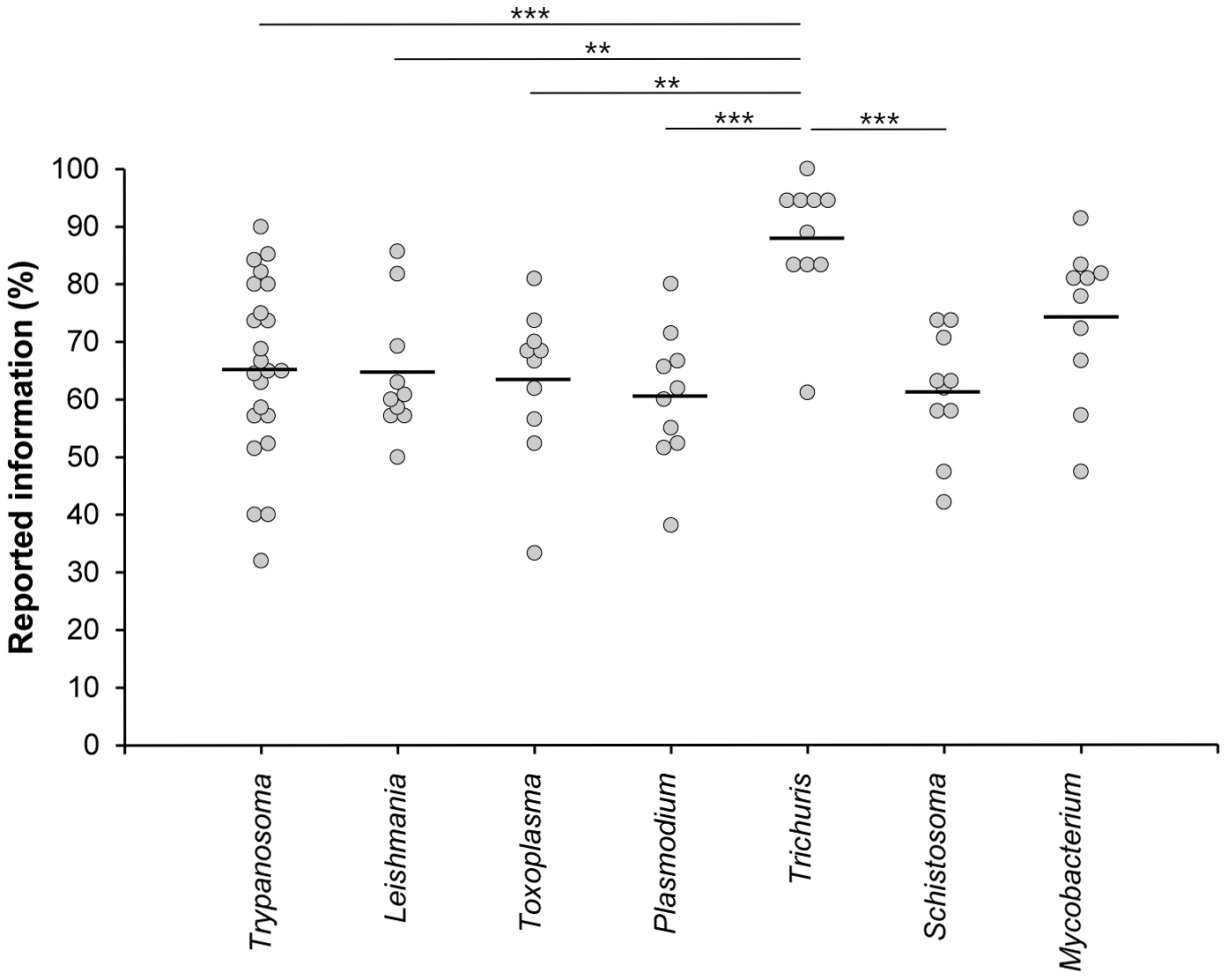
Box-percentile plot to compare the quality of methods reporting in parasitology experiments. Articles about “Trypanosomiasis”[MeSH]; “Leishmaniasis”[MeSH]; “Toxoplasmosis”[MeSH]; “Malaria”[MeSH]; “Trichuris”[MeSH]; “Schistosoma”[MeSH] and “Tuberculosis”[MeSH]. The figure shows that the experimental model of colitis induced by *Trichuris* had the highest scores, followed by tuberculosis, *Trypanosoma*, *Toxoplasma*, *Leishmania*, *Plasmodium* and *Schistosoma* experiments. P values less than 0.01 and 0.001 are represented by ** and *** respectively.

In order to test whether our observations about the quality of method reporting were a general phenomenon or whether they were specific to trypanosomiasis we evaluated 10 articles each on *Leishmania*, *Toxoplasma* and *Plasmodium*; these diseases were chosen because they are also complex and considered public health issues. As in the articles about *Trypanosoma* experiments, no article about *Leishmania*, *Toxoplasma* and *Plasmodium* experiments met all criteria that should be reported on our checklist, although one publication on *Leishmania* [49] scored 100% for the parasite and host domains (Table S5 and S6). There was no significant difference in the percentage of reported information between *Trypanosoma*, *Leishmania*, *Toxoplasma* and *Plasmodium* experiments (Figure 6). The lowest scores were found in the host domain in *Leishmania* and *Toxoplasma* experiments (20%, Table S5). *Plasmodium* experiments had the lowest score in the parasite domain (25%, Table S6) and *Leishmania* had the lowest score in the experimental infection domain (30%, Table S7). No *Toxoplasma* or *Plasmodium* experiment met all of the criteria in any domain (Table S5, S6 and S7).

In contrast to all protozoan parasite experiments, the quality of method reporting in the helminth model of infection by *Trichuris muris* showed the highest scores in all three domains (Figure 6). One *Trichuris muris* experiment [83] successfully scored 100% in all three domains. *Trichuris muris* experiments reported significantly more information than *Trypanosoma* (*p*<0.001), *Plasmodium* (*p*<0.001), *Schistosoma* (*p*<0.001), *Leishmania* (*p*<0.01) and *Toxoplasma* (*p*<0.01) experiments (Figure 6). However, the other helminth model, *Schistosoma sp*., scored poorly with the second lowest mean reported information (61.16%). *Mycobacterium* (mean reported information 73.96%), the non-parasitic bacterial infection model, scored more highly than *Trypanosoma* (mean reported information 65.46%) but this was not significant.

### Validation of scoring methods

**Figure 7:**
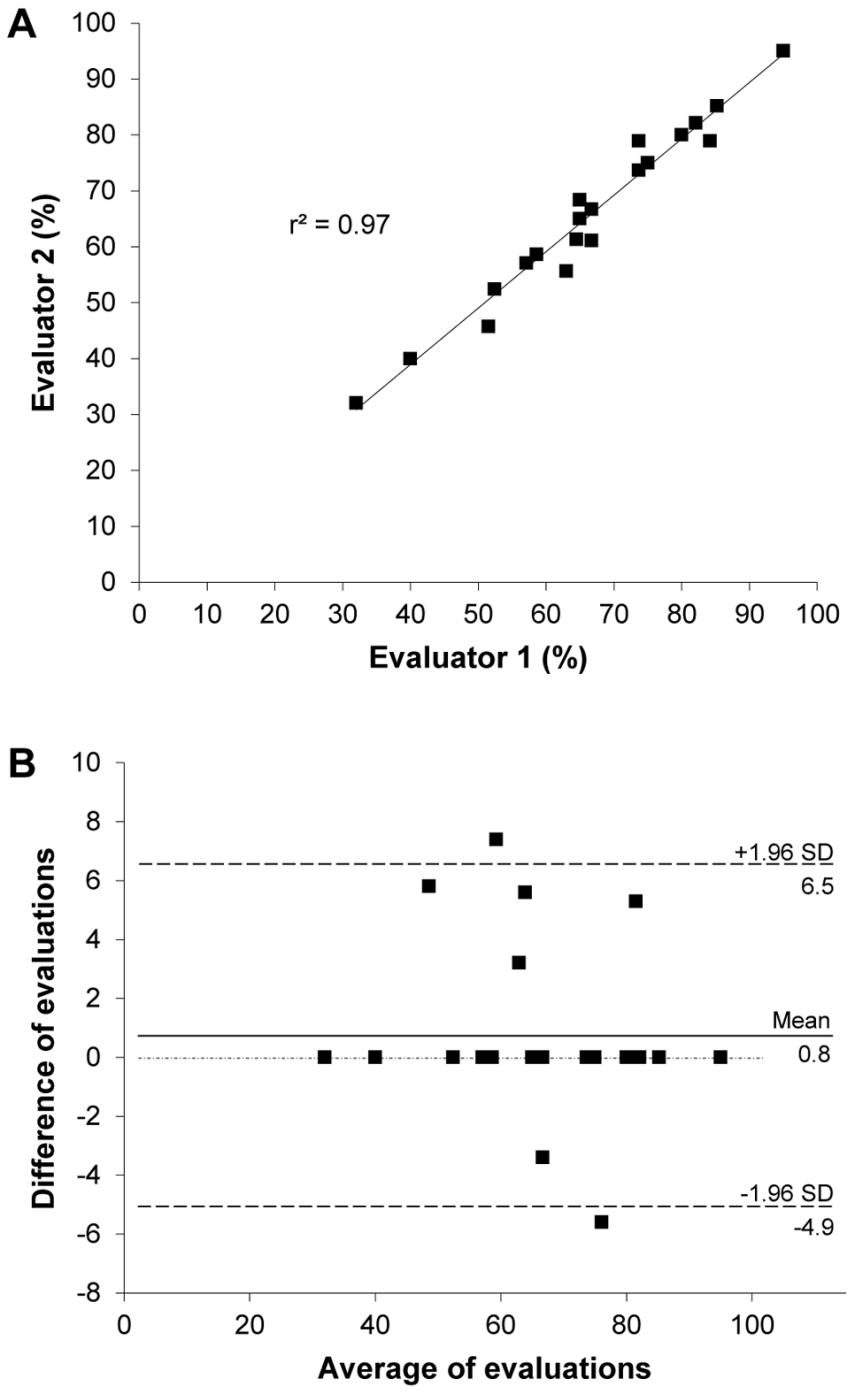
Linear correlation (A) and Bland-Altman (B) plots between scores of method reporting in *Trypanosoma* experiments. Evaluation based strictly on what was explicitly included in the published paper (Evaluator 1) and on interpretations and assumptions determined by an expert in the field (Evaluator 2).

The papers from *Trypanosoma* experiments were initially scored by the first and second authors. A specialist in trypanosomiasis then independently scored these papers. The evaluation made by the trypanosomiasis specialist scored 61.6% for the number of criteria from the checklist met in the corpus of articles, whereas a strict evaluation scored 65%. These evaluations scored 63.8% and 64.9% respectively after reviewing the results of both examinations. A linear correlation test (Figure 7A) showed a strong and significant linear correlation between the scores (r^2^ = 0.96; *p*<0.0001); suggesting that the checklist items measure a common domain and that the personal opinion of the coder does not have an important impact on the scores. In addition, a Bland-Altman test (Figure 7B) was used to verify the agreement between the two evaluations. This analysis showed a good concordance as 16 points were on the line of no difference and 21 fell within the 95% limits of agreement (mean  = 0.80 and SD: ±2.91), verifying that the scoring was consistent between the evaluators.

## Discussion

In order to draw conclusions about the quality of method information reported in articles and its impact on the replicability, reproducibility and comparability of experimental work, we have selected trypanosome infection models as a focus of study. Trypanosomiasis as a complex disease is an appropriate example to understand the importance of the subtlety of experimental variables in the outcome of the modelled disease. Our results indicate that the quality of method information reported in articles about experimental infection with *Trypanosoma spp* is a cause for concern and it has not shown improvement over time, despite there being evidence that most of these variables do influence the results.

Many studies have demonstrated the genetic diversity of *Trypanosoma* species [108], [109], as well as the diversity of outcome associated with different parasite strains [17]. The classically described differences in humans infected with different subspecies of *T. brucei* or lineages of *T. cruzi* are well recognized. *T. brucei rhodesiense* causes acute disease and *T. brucei gambiense* causes a more chronic infection [110]. *T. b. gambiense* is divided into two groups which differ in phenotype including pathology [111]. In addition, the cardiomyopathy and digestive forms of Chagas' disease have been associated with *T. cruzi* lineage I and *T. cruzi* lineage II respectively [112]. Strain differences have also been observed in the three major strains of *Toxoplasma*, which vary greatly in their virulence and infection outcome [113]. In addition, isolates of *Trichuris muris* not only differ in virulence but can also trigger changes in the immune response elicited in susceptible hosts [114]; whereas eggs from different strains of *Schistosoma mansoni* cause specific granulomatous responses [115]. Consequently, reporting genus and species of the parasite is not enough; the parasite strain must be reported and if the parasite is a new isolate, it should be characterized.

Virulence of the parasite in all stages of its life cycle plays an important role in the outcome of infection. For example, the failure of laboratory experiments to develop successful malaria vaccines has been attributed to the failure of models to include a skin stage, which is deemed integral to suppressing host immunity and initiating tolerance to the parasite [116]. In *T. cruzi*, several factors have been implicated in the formation of the infective metacyclic stages. Long-term axenic cultures of *T. cruzi* exhibit a lower capacity to transform into metacyclic trypomastigotes, in comparison to those maintained by alternate invertebrate/vertebrate passages [117]. In addition, the infectivity of *T. cruzi* clones is modified when it is grown in different hosts; a clone passaged through mice has been shown to be more virulent to mice and guinea pigs than the same clone passaged through guinea pigs, the virulence of which remained unchanged [118]. Infection route has also been shown to exert significant impact on the overall course and outcome of infection. In Chagas disease, for instance, the outbreaks associated with food/beverage consumption display severe clinical features in comparison with those of patients that have been infected with *T. cruzi* by vector transmission [119]; a phenomenon that has been associated with the sylvatic biodemes and genotypes of *T. cruzi* [120], [121]. In addition, in *Toxoplasma* infections, mice may be susceptible or resistant to infection depending on whether an oral or intraperitoneal challenge is used [122].

Since gender and the corresponding sex steroids affect the immune response [123], [124] it is important to specify the gender of experimental animals used. Sex-differences have been demonstrated previously in several experimental infections. For example, in BALB/k mice, males are more resistant to *Toxoplasma gondii* than females [125]. Conversely, in BALB/c mice lacking IL-4, and C57BL/6 p55^−/−^ or p75^−/−^ mice, it is the female mice that are better at expelling *Trichuris muris* than males [84]. However, only 70% of *Trypanosoma* studies reported the sex of animals used in the experimental infection and only 25% reported the gender of animals used to maintain parasite stocks (Tables S1 and S2). In experimental trypanosome infections a gender-related effect has been shown: using BALB/c mice infected with a natural dose of vector-derived metacyclic trypomastigotes of *T. cruzi* (100 parasites/mouse) the peak of parasitaemia in males was about four-fold higher than that in females [126]. Similarly, an experimental infection with a strain of *T. brucei brucei* at 50% of the mouse lethal dose showed that the female were more trypanotolerant than the males and there was no evidence that this was X-linked [127], [128]. Housing conditions and social environment also affect the course of experimental trypanosome infections. For example, the parasitaemia levels vary according to whether the animals are kept individually or in a group due to pheromones of the opposite sex [126], [129]. Furthermore, hormonal profiles during the oestrous cycle are not only modified by the parasite; such as *T. congolense* [130], but also by the light/dark cycle conditions [131].

In the case of contradictory results, the reporting of the essential parameters that describe a parasitic experimental infection can help to determine the nature of their discrepancies. To exemplify this issue, we have chosen two papers published in the journal Infection and Immunity that were undertaken to assess the role of Nitric Oxide (NO) in immunity to *T. cruzi* infection and their experiments showed contradictory results. Vespa *et al*. claim that NO is involved in control of *T. cruzi*-induced parasitaemia [20], whereas Cummings *et al*. claim that NO is not required for control of *T. cruzi* in the acute or chronic stages of the infection [23]. However, although these studies were carried out using female mice on a C57BL/6 background, the experimental infections were performed using different *T. cruzi* strains, which could explain, at least in part, the differences in their findings: mice infected with 10^4^ trypomastigotes of the Y strain showed peak parasitaemia at day 8 that decreased thereafter [20], whereas mice infected with 10^3^ trypomastigotes of the Brazil strain showed a peak at day 30 and decreased thereafter [23]. Moreover, although both infections were performed with blood-derived trypomastigotes none of them reported species, gender and age of the animals used to culture the parasite; important parameters that modified the infectivity of *T. cruzi* [117], [118]. In addition, there is experimental evidence that shows significant differences among parasitaemia curves between older and younger BALB/c mice infected with a long-term mouse-passaged clone of the *T. cruzi* isolate TolAc1; higher parasitaemia levels were observed in older animals (31-day-old) with lower inoculum (3×10^4^ trypomastigotes) than younger animals (8-day-old) with higher inoculum (9×10^4^ trypomastigotes) [118]. However, the age of the animals used to evaluate the role of NO in the control of *T. cruzi* infection was reported by Vespa *et al*. but not by Cummings *et al*. [20], [23]. Thus, these and other conditions that could also influence the parasitaemia and, hence, the researched outcome should be reported in order to understand the complexity of these parasitoses.

Although the information collected through the checklist should be reported for all *Trypanosoma* experiments, some information could be inferred from the characteristics of the experimental processes, although this depends on the level of expertise of observers (i.e. non-experts and experts). In this way, a factor such as the stage of the parasite used for a *T. cruzi* infection could be easily inferred by an expert since he/she knows that the infectious stage is the trypomastigote. Moreover, both experts and non-experts could also infer many details of the conditions used in cell cultures by assuming experimenters have opted for the most commonly used parameters. For example temperature and CO_2_ atmosphere are usually set to 37°C and 5% of CO_2_. However, neither experts nor non-experts could infer the species and strain of the parasite; age and gender of the host; or the inoculum used in the infection assays, among others. Thus the validation of data becomes a difficult or impossible task when there is not only not enough information about the method used, but also most of the missing information cannot be inferred, even by an expert.

Providing a high-quality description of the experimental method is important not only to replicate and reproduce, but also to compare and integrate that data and, hence, facilitate translational discoveries. The issues found in reporting methods probably stem, at least in part, from the current structure of scientific publishing, which is not adequate to effectively communicate complex experimental methods. This problem has been recognised, with some journals already introducing editorial measures and methods checklists in order to improve the quality of methods reporting [132].

For the field of trypanosomiasis we have created a checklist to guide parasitologists in reporting *Trypanosoma* experiments (see Annex 1). This checklist included the minimum information that should be provided when describing the parasite, host and infection aspects of those experiments. Our checklist does not cover aspects inherent to each possible experimental assay such as those derived from omics and conventional technologies. In these cases, the BioSharing catalogue [133] should be consulted for checklists: e.g. the Minimum Information About a Microarray Experiment (MIAME) and Proteomics Experiment (MIAPE); and the Minimum Information for Publication of Quantitative Real-Time PCR Experiments (MIQE). Moreover, there are other guidelines such as the Minimum Information About a Cellular Assay (MIACA) and the Animals in Research: Reporting *In Vivo* Experiments (ARRIVE) that provide detailed descriptions of experiments performed on cell and animal models.

In conclusion, it has become clear that biomedical science is plagued by findings that cannot be reproduced and/or compared; and the parasitology community is no stranger to this, as has been shown by this study. Nevertheless, the scientific community that works on trypanosomiases is small and many of them know each other personally so in principle it should be possible to change the way that *Trypanosoma* experiments are reported. However, it is important that the scientific community as a whole is engaged with that process. Finally, the checklist has been demonstrated to be applicable to several different infection models and could be implemented to improve the quality of methods reporting for all infection experiments in principle.

## Materials and Methods

### Search strategy

The method of the literature review follows the recommendations outlined in the PRISMA guidelines [134]. A protocol was designed to identify the method information reported in published articles that utilised experimental infection with *Trypanosoma* species, where the effects on gene expression –transcriptomics and proteomics– of the host were studied. Criteria in three domains were evaluated: characteristics and culture conditions of the parasite, characteristics and maintenance conditions of the host and the infection procedure. The protocol used here for capturing data has not been previously published.

The literature search was conducted using Medline via PubMed. The database was searched in April 2013 for articles that were published between 1^st^ January, 2000 and 31^st^ December, 2012 using the MeSH (Medical Subject Headings) terms as they appear in Table 2. The PubMed Identifier (PMID) numbers were used to identify those articles that were common between “Genes” AND “Trypanosomiasis” and “Proteins” AND “Trypanosomiasis”. The search was not limited by study design or by language of publication. The year 2000 was chosen because it was the year in which the first rough draft of the human genome was completed [135], [136] and these data were used in many fields of medicine including infectious disease. In addition, we chose to focus on gene expression profiling in the host due to an experimental *Trypanosoma* infection because it provides the broadest evidence about the molecular physiopathology of trypanosomiasis.

**Table 2:** Search terms used in PubMed.

Search	Terms
Search 1	“Genes”[MeSH] AND “Trypanosomiasis”[MeSH]
Search 2	“Proteins”[MeSH] AND “Trypanosomiasis”[MeSH]
Search 3	“Microarray Analysis”[MeSH] AND “Trypanosomiasis”[MeSH]
Search 4	“Proteomics”[MeSH] AND “Trypanosomiasis”[MeSH]

In order to compare the quality of method reporting in *Trypanosoma* experiments with the reporting of other parasitic disease infections we collected a subset of *Leishmania*, *Toxoplasma* and *Plasmodium* experimental infection models, since diseases produced by them are also complex and considered public health issues. In addition, as a comparative control of methods reporting in experimental infections, we sought two models of worm infection: one with a simple life cycle (*Trichuris muris*) and another with a complex life cycle (*Schistosoma sp*.); requiring adaptation for survival in fresh water as free-living forms and as parasites in snail intermediate and vertebrate definitive hosts. In addition, we assessed tuberculosis infectious models in order to have a general idea about the quality of method reporting in non-parasitic infection models. Tuberculosis was chosen because it is probably one of the most studied infectious disease.

The same search strategy was carried out where the MeSH term “Trypanosomiasis” was replaced with the following MeSH terms: “Leishmaniasis”, “Toxoplasmosis”, “Malaria”, “*Trichuris*”, “*Schistosoma*” and “Tuberculosis”. To avoid selection bias, the articles were randomly ordered and the first 10 articles for each extra parasitosis and the non-parasitic infection model (*Mycobacterium*) that described gene expression profiling in the host due to an experimental infection were selected.

Study selection was made by one reviewer and checked independently by a second reviewer, any disagreement was resolved by consensus or by discussion with a third reviewer. Only primary research papers were included in the search. The titles and abstracts of articles were reviewed and analysed in detail to filter out those in which the experiments were performed on the parasite or on vector insects and keep those done on the host. This corpus of articles was then used to confirm eligibility and to extract data.

### Structure definition and data extraction

A checklist that contains the minimum information required about the parasite, host and infection to describe an experiment carried out with any *Trypanosoma* species was elaborated by experts in the field of trypanosomiasis research and it is presented in Table 3. Pre-analytical variables in the methods were prioritised in this list because they are critical for interpretation of the results. The terms were classified into three domains according to their roles in a *Trypanosoma* experiment: the host, the parasite and the infection. A data extraction sheet was developed to annotate the information reported in the methods and results sections. Data extraction and quality assessment were carried out by one author and checked by a second reviewer, and inconsistencies were discussed by both reviewers and consensus reached.

**Table 3:** Checklist for the reporting of *Trypanosoma* experiments.

Topic	Item#	Description	Does it meet?
**Parasite information**			
General	1	Identify the species of the parasite	
	2	Identify the strain of the parasite	
	3	Identify the stage of the parasite used	
Culture conditions for parasites grown *in vivo*	4	Identify the species and strain of the animal	
	5	Describe the age of the animal	
	6	Describe the gender of the animal	
	7	Identify the parasite collection sample	
Culture conditions for parasites grown *in vitro*	8	Identify the cell type	
	9	Describe the culture medium used	
	10	Describe the supplements and antibiotics used	
	11	Describe the temperature and CO_2_ atmosphere of the culture	
Time of growing	12	Describe the time of growing of the parasite prior to infection	
**Host information**			
Animals	13	Identify the species and strain of the animal	
	14	Describe the age of the animal	
	15	Describe the gender of the animal	
	16	Describe the housing conditions (light/dark cycle)	
	17	Describe the method of sacrifice	
Cell	18	Identify the cell type	
	19	In primary culture, identify the organ/tissue from which cells come	
	20	In primary culture, describe the method of purification of the cells	
	21	Describe the culture medium used	
	22	Describe the supplements and antibiotics used	
	23	Describe the temperature and CO_2_ atmosphere of the culture	
	24	Describe the time of growing of the cells prior to infection	
**Experimental infection information**			
Animal	25	Describe the inoculum –parasites per animal- used	
	26	Describe the way of inoculation	
	27	Describe the medium of inoculation	
	28	Report the parasitaemia and the time in which the parasitaemia was measured	
	29	Report the mortality of the animals post-infection	
Cell	30	Report the purity of the primary culture	
	31	Report the viability of cells prior infection	
	32	Describe the ratio –parasites per cell- used	
	33	Report the percentage of infected cells	
Parasite	34	Report the viability of parasites prior infection	
	35	Describe the purity of infective forms of the parasite	
	36	Describe the time course (length) of infection	

### Bibliometric indices

Bibliometric parameters were used to determine if they were associated with the quality of method reporting. The impact factor (IF) of each journal was retrieved from the Institute for Scientific Information (ISI) Web of Knowledge's Journal Citation Reports database science edition 2011. The number of citations was measured by the total recorded for each article by Thomson Scientific's Web of Science and Google Scholar in May 2013. For each corresponding author, the h-index was obtained through Thomson Scientific's Web of Science using a citation window up to one year before the article was published. The h-index was searched in two different ways: first, using the full name of the corresponding author and second, filtering the result by topic, using the term “Trypanosom*”. The number of articles published for each journal about trypanosomiasis was sought in PubMed using the short name of the journals and the MeSH term “Trypanosomiasis”. The search was filtered by time; from 1^st^ January, 2000 to 31^st^ December, 2012.

### Validity of scoring methods

An expert in trypanosomiasis tested the quality of reported information on *Trypanosoma* experiments. The expert scored the corpus of articles using the checklist that contains the minimum information required to describe a *Trypanosoma* experiment (Table 3). This evaluation was based strictly on what was explicitly included in the published paper and its results are presented throughout this article. The validity of this assessment was tested based on its agreement with another evaluation based on interpretations and assumptions determined by another expert in the field in order to avoid bias of the retrieval results by interpretation.

### Statistical analysis

For each article, the percentage of reported information in each article domain was obtained by direct counting. Linear and Spearman's rank correlations and Bland-Altman comparison were calculated using STATA software [137] and the equivalence of between scores obtained by the evaluators was determined by a correlation test. Comparisons between experimental infection models were performed using a one-way ANOVA in GraphPad PRISM 4 software [138].

## Supporting Information

Table S1 Quality measures of the studies that failed to fulfil any one of data of minimal information about the parasite in *Trypanosoma* experiments.
(PDF)

Table S2 Quality measures of the studies that failed to fulfil any one of data of minimal information about the host in *Trypanosoma* experiments.
(PDF)

Table S3 Quality measures of the studies that failed to fulfil any one of data of minimal information about the experimental infection in *Trypanosoma* experiments.
(PDF)

Table S4 Bibliometric indices in reporting *Trypanosoma* experiments.
(PDF)

Table S5 Quality measures of the studies that failed to supply any one of the criteria for minimal information about the parasite in *Leishmania*, *Toxoplasma*, *Plasmodium*, *Trichuris*, *Schistosoma* and *Mycobacterium* experiments.
(PDF)

Table S6 Quality measures of the studies that failed to supply any one of the criteria for minimal information about the host in *Leishmania*, *Toxoplasma*, *Plasmodium*, *Trichuris*, *Schistosoma* and *Mycobacterium* experiments.
(PDF)

Table S7 Quality measures of the studies that failed to supply any one of the criteria for minimal information about the experimental infection in *Leishmania*, *Toxoplasma*, *Plasmodium*, *Trichuris*, *Schistosoma* and *Mycobacterium* experiments.
(PDF)

Checklist S1 PRISMA Checklist.
(DOC)
