# Supplementary material for: The Quality of Methods Reporting in Parasitology Experiments
Source: PLoS One. 2014 Jul 30;9(7):e101131. doi: 10.1371/journal.pone.0101131 (PMC4116335; doi:10.1371/journal.pone.0101131)
Supplement: Table S1 — Quality measures of the studies that failed to fulfil any one of data of minimal information about the parasite in Trypanosoma experiments. (PDF) [file pone.0101131.s001.pdf]

Table S1. Quality measures of the studies that failed to fulfil any one of data of minimal information about the parasite in *Trypanosoma* experiments.

				**Culture conditions of Trypanosomes**										
	**Parasite information**			**Parasites from animals**				**Parasites from cells**						
**Articles**	**P1**	**P2**	**P3**	**P4**	**P5**	**P6**	**P7**	**P8**	**P9**	**P10**	**P11**	**P12**	**Total**	**%**
Amin et al., 2010	✓	✓	NA	NA	NA	NA	NA	NA	NA	NA	NA	NA	2/12	16.7%
Chessler et al., 2009	✓	NA	✓	*	*	*	*	✓	✓	✓	✓	NA	6/8	75%
Costales et al., 2009	✓	✓	✓	*	*	*	*	✓	✓	✓	✓	NA	7/8	87.5%
Garg at al., 2004	✓	✓	✓	*	*	*	*	✓	✓	✓	NA	NA	6/8	75%
Genovesio et al., 2011	✓	✓	✓	*	*	*	*	✓	✓	✓	✓	NA	7/8	87.5%
Goldenberg et al., 2009	✓	✓	✓	*	*	*	*	✓	✓	✓	✓	✓	8/8	100%
Hashimoto et al., 2005	✓	✓	✓	*	*	*	*	✓	✓	✓	✓	✓	8/8	100%
Hill et al., 2005	✓	✓	✓	✓	*	*	*	*	*	*	*	*	4/4	100%
Kierstein et al., 2006	✓	✓	✓	✓	NA	NA	✓	*	*	*	*	*	5/7	71.4%
Li et al., 2009	✓	✓	✓	✓	✓	✓	✓	*	*	*	*	*	7/7	100%
Li et al., 2011	✓	✓	✓	✓	✓	✓	✓	*	*	*	*	*	7/7	100%
Lopez et al., 2008	✓	✓	✓	✓	✓	✓	✓	*	*	*	*	*	7/7	100%
Manque et al., 2011	✓	✓	✓	*	*	*	*	✓	NA	NA	NA	NA	4/8	50%
Meade et al., 2009	✓	NA	✓	✓	*	*	*	*	*	*	*	*	3/4	75%
Mekata et al., 2012	✓	✓	NA	NA	NA	NA	NA	NA	NA	NA	NA	NA	1/12	8.3%
Mukherjee et al., 2003	✓	✓	✓	NA	NA	NA	NA	NA	NA	NA	NA	NA	3/12	25%
Mukherjee et al., 2008	✓	✓	✓	NA	NA	NA	NA	NA	NA	NA	NA	NA	3/12	25%
Noyes et al., 2009	✓	✓	✓	✓	✓	NA	✓	*	*	*	*	*	6/7	85.7%
O'Gorman et al., 2009	✓	✓	✓	✓	NA	NA	*	*	*	*	*	*	4/6	66.7%
Soares et al., 2010	✓	✓	✓	*	*	*	*	✓	NA	NA	NA	NA	4/8	50%
Soares et al., 2011	✓	✓	✓	*	*	*	*	✓	NA	NA	NA	NA	4/8	50%
Graefe et al., 2006	✓	✓	✓	✓	NA	NA	NA	*	*	*	*	*	4/7	57.1%
Tanowitz et al., 2011	✓	✓	✓	✓	NA	NA	✓	✓	✓	✓	✓	✓	10/12	83.3%
Total	23/23	21/23	21/23	10/14	4/12	3/12	6/11	10/14	7/14	7/14	6/14	3/14		
%	100%	91.3%	91.3%	71.4%	33.3%	25%	54.5%	71.4%	50%	50%	42.9%	21.4%		

Criteria: P1 (species), P2 (strain), P3 (stage), P4 (species and strain of animal), P5 (age), P6 (gender), P7 (parasite collection sample); P8 (cell type), P9 (culture medium), P10 (supplements and antibiotics), P11 (temperature and CO_2 atmosphere), and P12 (time of growing of the parasite prior to infection).

✓: meets the criteria

NA: information not available

*****: not applicable
