# Supplementary material for: The Quality of Methods Reporting in Parasitology Experiments
Source: PLoS One. 2014 Jul 30;9(7):e101131. doi: 10.1371/journal.pone.0101131 (PMC4116335; doi:10.1371/journal.pone.0101131)
Supplement: Table S2 — Quality measures of the studies that failed to fulfil any one of data of minimal information about the host in Trypanosoma experiments. (PDF) [file pone.0101131.s002.pdf]

Table S2. Quality measures of the studies that failed to fulfil any one of data of minimal information about the host in *Trypanosoma* experiments.

	Characteristics and culture conditions of the host models													
	Animal model					Cell model								
Articles	H1	H2	H3	H4	H5	H6	H7	H8	H9	H10	H11	H12	Total	%
Amin et al., 2010	✓	✓	NA	NA	✓	*	*	*	*	*	*	*	3/5	60%
Chessler et al., 2009	✓	NA	NA	NA	NA	✓	✓	✓	✓	*	*	NA	5/10	50%
Costales et al., 2009	*	*	*	*	*	✓	✓	✓	✓	*	*	✓	5/5	100%
Garg at al., 2004	✓	✓	✓	NA	NA	*	*	*	*	*	*	*	3/5	60%
Genovesio et al., 2011	*	*	*	*	*	✓	✓	✓	✓	*	*	✓	5/5	100%
Goldenberg et al., 2009	✓	✓	✓	NA	✓	✓	✓	✓	✓	✓	✓	✓	11/12	91.7%
Graefe et al., 2006	✓	✓	NA	NA	NA	*	*	*	*	*	*	*	2/5	40%
Hashimoto et al., 2005	*	*	*	*	*	✓	✓	✓	✓	*	*	✓	5/5	100%
Hill et al., 2005	✓	✓	✓	NA	*	*	*	*	*	*	*	*	3/4	75%
Kierstein et al., 2006	✓	✓	NA	NA	NA	*	*	*	*	*	*	*	2/5	40%
Li et al., 2009	✓	✓	NA	✓	✓	*	*	*	*	*	*	*	4/5	80%
Li et al., 2011	✓	✓	✓	✓	✓	*	*	*	*	*	*	*	5/5	100%
Lopez et al., 2008	✓	✓	✓	NA	NA	✓	✓	✓	✓	✓	✓	*	9/11	81.8%
Manque et al., 2011	✓	✓	✓	NA	NA	✓	✓	✓	✓	✓	✓	✓	10/12	83.3%
Meade et al., 2009	✓	✓	✓	NA	*	*	*	*	*	*	*	*	3/4	75%
Mekata et al., 2012	✓	✓	✓	NA	NA	*	*	*	*	*	*	*	3/5	60%
Mukherjee et al., 2003	✓	✓	✓	NA	NA	*	*	*	*	*	*	*	3/5	60%
Mukherjee et al., 2008	✓	✓	✓	NA	NA	✓	✓	✓	✓	✓	✓	*	9/11	81.2%
Noyes et al., 2009	✓	✓	NA	NA	✓	*	*	*	*	*	*	*	3/5	60%
O'Gorman et al., 2009	✓	✓	✓	NA	*	*	*	*	*	*	*	*	3/4	75%
Soares et al., 2010	✓	NA	✓	NA	NA	*	*	*	*	*	*	*	2/5	40%
Soares et al., 2011	✓	✓	✓	✓	NA	*	*	*	*	*	*	*	4/5	80%
Tanowitz et al., 2011	✓	NA	✓	NA	NA	✓	✓	✓	✓	✓	✓	NA	8/12	66.7%
Total	20/20	17/20	14/20	3/20	5/17	9/9	9/9	9/9	9/9	5/5	5/5	5/7		
%	100%	85%	75%	15%	29.4%	100%	100%	100%	100%	100%	100%	71.4%		

Criteria: H1 (species and strain), H2 (age); H3 (gender), H4 (light and dark cycle), H5 (method of sacrifice), H6 (cell type), H7 (culture medium), H8 (supplements and antibiotics), H9 (temperature and CO_2 atmosphere), H10 (organ or tissue which takes the primary culture), H11 (method of purification for establishing primary culture), and H12 (time of growing of the cell prior infection).

✓: meets the criteria

NA: information not available

*****: not applicable
