# Supplementary material for: The Quality of Methods Reporting in Parasitology Experiments
Source: PLoS One. 2014 Jul 30;9(7):e101131. doi: 10.1371/journal.pone.0101131 (PMC4116335; doi:10.1371/journal.pone.0101131)
Supplement: Table S3 — Quality measures of the studies that failed to fulfil any one of data of minimal information about the experimental infection in Trypanosoma experiments. (PDF) [file pone.0101131.s003.pdf]

Table S3. Quality measures of the studies that failed to fulfil any one of data of minimal information about the experimental infection in *Trypanosoma* experiments.

	Characteristics of the experiment													
	Animal models					Cellular models				Parasite				
**Articles**	**I1**	**I2**	**I3**	**I4**	**I5**	**I6**	**I7**	**I8**	**I9**	**I10**	**I11**	**I12**	**Total**	**%**
Meade et al., 2009	✓	✓	✓	NA	NA	*	*	*	*	NA	✓	✓	5/8	62.5%
Amin et al., 2010	✓	✓	NA	NA	NA	*	*	*	*	NA	NA	✓	3/8	37.5%
Chessler et al., 2009	✓	✓	✓	✓	NA	*	NA	✓	NA	NA	NA	✓	6/11	54.5%
Costales et al., 2009	*	*	*	*	*	*	NA	NA	✓	NA	NA	✓	2/6	33.3%
Garg at al., 2004	✓	✓	NA	NA	NA	*	*	*	*	NA	NA	✓	3/8	37.5%
Genovesio et al., 2011	*	*	*	*	*	*	NA	✓	NA	NA	NA	✓	3/6	33.3%
Goldenberg et al., 2009	*	*	*	*	*	✓	NA	✓	✓	NA	NA	✓	4/7	57.1%
Graefe et al., 2006	✓	✓	✓	✓	✓	*	*	*	*	NA	✓	✓	7/8	87.5%
Hashimoto et al., 2005	*	*	*	*	*	*	NA	✓	✓	NA	NA	✓	3/6	50%
Hill et al., 2005	✓	✓	✓	NA	NA	*	*	*	*	NA	✓	✓	4/6	62.5%
Kierstein et al., 2006	✓	✓	✓	✓	NA	*	*	*	*	NA	✓	✓	6/8	75%
Li et al., 2009	✓	✓	✓	✓	✓	*	*	*	*	NA	✓	✓	7/8	87.5%
Li et al., 2011	✓	✓	NA	NA	NA	*	*	*	*	NA	✓	✓	4/8	50%
Lopez et al., 2008	✓	✓	✓	✓	✓	NA	NA	*	*	NA	✓	✓	7/10	70%
Manque et al., 2011	*	*	*	*	*	NA	NA	✓	✓	NA	NA	✓	3/7	42.9%
Mekata et al., 2012	✓	✓	NA	✓	✓	*	*	*	*	NA	NA	✓	5/8	62.5%
Mukherjee et al., 2003	✓	NA	NA	✓	✓	*	*	*	*	NA	NA	✓	4/8	50%
Mukherjee et al., 2008	✓	✓	NA	NA	✓	✓	NA	*	*	NA	NA	✓	5/10	50%
Noyes et al., 2009	✓	✓	✓	✓	✓	*	*	*	*	NA	✓	✓	7/8	87.5%
O'Gorman et al., 2009	✓	✓	✓	NA	NA	*	*	*	*	NA	✓	✓	5/8	62.5%
Soares et al., 2010	✓	✓	NA	✓	✓	*	*	*	*	NA	NA	✓	5/8	62.5%
Soares et al., 2011	✓	✓	✓	NA	NA	*	*	*	*	NA	NA	✓	4/8	50%
Tanowitz et al., 2011	*	*	*	*	*	NA	NA	✓	NA	NA	NA	✓	2/7	28.6%
Total	17/17	16/17	10/17	9/17	8/17	2/5	0/9	6/7	4/7	0/23	9/23	23/23		
%	100%	94.1%	58.8%	52.9%	47.1%	40%	0%	85.7%	57.1%	0%	39.1%	100%		

Criteria: I1 (inoculum –parasite per animal), I2 (route of inoculation), I3 (medium of inoculation), I4 (parasitaemia and time post infection when parasitaemia was measured), I5 (mortality of animals post infection), I6 (purity of primary culture), I7 (viability of the cells prior to infection), I8 (ratio –parasites per cell), I9 (percentage infected cells), I10 (viability of the parasite prior to infection), I11 (purity of the infective form of the parasite), and I12 (duration of infection).

✓: meets the criteria

NA: information not available

*****: not applicable
