# Supplementary material for: The Quality of Methods Reporting in Parasitology Experiments
Source: PLoS One. 2014 Jul 30;9(7):e101131. doi: 10.1371/journal.pone.0101131 (PMC4116335; doi:10.1371/journal.pone.0101131)
Supplement: Table S4 — Bibliometric indices in reporting Trypanosoma experiments. (PDF) [file pone.0101131.s004.pdf]

Table S4. Bibliometric indices in reporting *Trypanosoma* experiments.

Journal			Citations			Author		
Name	IF	Topic	Reference	Web of Science	Google Scholar	Corresponding author	h-index	h-index*
Am J Trop Med Hyg	2.592	172	Amin et al., 2010	13	17	Daniel N. Amin	3	3
Biochem J	4.897	6	Garg at al., 2004	20	19	N. Garg	6	5
BMC Genomics	4.073	6	Costales et al., 2009	7	12	Barbara A. Burleigh	14	13
BMC Genomics	4.073	6	O'Gorman et al., 2009	8	9	David E. MacHugh	13	2
Cell Cycle	5.359	125	Soares et al., 2011	10	13	Milena Soares	20	13
Cell Cycle	5.359	125	Tanowitz et al., 2011	0	1	H. B. Tanowitz	33	20
Exp Parasitol	2.122	150	Li et al., 2011	0	0	Zhao-Rong Lun	15	7
Genes Immun	3.872	3	Kierstein et al., 2006	10	12	S. Kierstein	1	1
Genomics	3.019	68	Mukherjee et al., 2008	10	16	H. B. Tanowitz	33	20
Infect Immun	4.165	93	Manque et al., 2011	11	14	Gregory A. Buck	20	12
Int J Parasitol	3.393	95	Hashimoto et al., 2005	2	2	J. Nakajima-Shimada	8	8
J Immunol	5.788	54	Chessler et al., 2009	8	11	Barbara A. Burleigh	14	13
J Immunol	5.788	54	Lopez et al., 2008	10	9	Donna M. Paulnock	12	5
J Infect Dis	6.410	60	Soares et al., 2010	11	15	Milena Soares	19	13
Microbes Infect	3.101	67	Goldenberg et al., 2009	6	8	David Spray	48	5
Mol Immunol	2.897	10	Meade et al., 2009	1	3	David E. MacHugh	13	2
Parasite Immunol	2.601	35	Mekata et al., 2012	0	0	Kazuhiko Ohashi	23	5
Parasitol Res	2.149	109	Li et al., 2009	3	4	Zhao-Rong Lun	14	5
Parasitol Res	2.149	109	Mukherjee et al., 2003	37	51	H. B. Tanowitz	25	13
PLoS One	4.092	54	Genovesio et al., 2011	6	9	Lucio H. Freitas-Junior	14	7
PLoS One	4.092	54	Graefe et al., 2006	3	5	Sebastian Graefe	5	3
PLoS One	4.092	54	Noyes et al., 2009	13	17	Jan Naessens	13	10
Vet Immunol Immunopathol	2.076	7	Hill et al., 2005	27	40	David E. MacHugh	9	2

Topic: articles published per journal about "Trypanosomiasis"[MeSH]
*****: h-index filtered by topic using the term Trypanosom*
