# Supplementary material for: The Quality of Methods Reporting in Parasitology Experiments
Source: PLoS One. 2014 Jul 30;9(7):e101131. doi: 10.1371/journal.pone.0101131 (PMC4116335; doi:10.1371/journal.pone.0101131)
Supplement: Table S5 — Quality measures of the studies that failed to supply any one of the criteria for minimal information about the parasite in Leishmania, Toxoplasma, Plasmodium, Trichuris, Schistosoma and Mycobacterium experiments. (PDF) [file pone.0101131.s005.pdf]

Table S5. Quality measures of the studies that failed to supply any one of the criteria for minimal information about the parasite in *Leishmania*, *Toxoplasma*, *Plasmodium*, *Trichuris*, *Schistosoma* and *Mycobacterium* experiments.

					**Culture conditions of *Leishmania, Toxoplasma, Plasmodium, Trichuris, Schistosoma* and *Mycobacterium***										
		**Parasite information**			**Parasites from animals**				**Parasites from cells**						
**Articles**	**Model**	**P1**	**P2**	**P3**	**P4**	**P5**	**P6**	**P7**	**P8**	**P9**	**P10**	**P11**	**P12**	**Total**	**%**
Park et al., 2000	L	✓	✓	✓	*	*	*	*	*	✓	✓	NA	NA	5/7	71.4%
Filippi et al., 2003	L	✓	✓	✓	*	*	*	*	*	✓	✓	NA	✓	6/7	85.7%
Bertholet et al., 2005	L	✓	✓	✓	*	*	*	*	*	✓	✓	NA	✓	6/7	85.7%
Kinjyo et al., 2006	L	✓	✓	✓	✓	NA	NA	NA	*	✓	✓	NA	NA	6/11	54.5%
Brunner et al., 2007	L	✓	✓	✓	✓	NA	NA	NA	*	✓	✓	✓	NA	7/11	63.6%
Guerfali et al., 2008	L	✓	✓	✓	*	*	*	*	*	✓	✓	✓	✓	7/7	100%
Jayakumar et al., 2008	L	✓	✓	✓	✓	NA	NA	✓	*	✓	✓	✓	✓	9/11	81.8%
Ehrchen at al., 2010	L	✓	✓	✓	*	*	*	*	*	✓	✓	NA	NA	5/7	71.4%
Biswas et al., 2011	L	✓	✓	✓	*	*	*	*	*	✓	✓	NA	NA	5/7	71.4%
de Carvalho et al., 2011	L	✓	✓	✓	*	*	*	*	*	✓	✓	✓	✓	7/7	100%
Desolme et al., 2000	T	✓	✓	✓	✓	NA	NA	✓	*	*	*	*	NA	5/8	62.5%
Gail et al., 2001	T	✓	✓	✓	*	*	*	*	✓	✓	✓	NA	NA	6/8	75%
Fux et al., 2003	T	✓	✓	✓	✓	NA	✓	✓	*	*	*	*	✓	7/8	87.5%
Tato et al., 2003	T	✓	✓	✓	✓	NA	NA	✓	*	*	*	*	NA	5/8	62.5%
Okomo et al, 2006	T	✓	✓	✓	*	*	*	*	✓	✓	✓	✓	NA	7/8	87.5%
Knight et al., 2006	T	✓	✓	✓	*	*	*	*	✓	✓	✓	NA	✓	7/8	87.5%
Watford et al., 2008	T	✓	✓	✓	NA	NA	NA	NA	*	*	*	*	NA	3/8	37.5%
Ju et al., 2009	T	✓	NA	✓	✓	NA	NA	✓	✓	✓	✓	NA	NA	7/12	58.3%
Fang et al., 2009	T	✓	✓	✓	✓	NA	NA	*	*	*	*	*	✓	5/7	71.4%
Zhuo et al., 2011	T	✓	✓	✓	NA	NA	NA	NA	*	*	*	*	NA	3/8	37.5%
Ylostalo et al., 2005	P	✓	NA	✓	✓	NA	NA	✓	*	*	*	*	NA	4/8	50%
Lovergrove et al., 2006	P	✓	✓	✓	✓	NA	NA	*	*	*	*	*	NA	4/7	57.1%
Delahaye et al., 2007	P	✓	✓	✓	✓	NA	NA	✓	*	*	*	*	✓	6/8	75%
Carapau et al., 2007	P	✓	NA	✓	NA	NA	NA	NA	*	*	*	*	NA	2/8	25%
Miu et al., 2008	P	✓	✓	✓	NA	NA	NA	NA	*	*	*	*	NA	3/8	37.5%
Randall et al., 2008	P	✓	✓	✓	NA	NA	NA	✓	*	*	*	*	NA	4/8	50%
Oakley et al., 2008	P	✓	✓	NA	NA	NA	NA	NA	*	*	*	*	NA	2/8	25%
Albuquerque et al, 2009	P	✓	✓	✓	✓	NA	NA	✓	*	*	*	*	NA	5/8	62.5%
Delic et al., 2011	P	✓	NA	✓	✓	NA	NA	NA	*	*	*	*	NA	3/8	37.5%
Rosanas et al., 2012	P	✓	✓	✓	NA	NA	NA	✓	*	*	*	*	NA	4/8	50%
Betts et al., 2001	C	✓	✓	✓	✓	✓	✓	*	*	*	*	*	✓	7/7	100%
Humphreys et al., 2004	C	✓	✓	✓	✓	✓	✓	*	*	*	*	*	✓	7/7	100%
Cliffe et al.,2005	C	✓	✓	✓	✓	✓	✓	*	*	*	*	*	✓	7/7	100%
Dixon et al., 2006	C	✓	✓	✓	✓	✓	✓	*	*	*	*	*	✓	7/7	100%
Bickle et al., 2007	C	✓	✓	✓	✓	✓	✓	*	*	*	*	*	✓	7/7	100%
Villarino et al., 2008	C	✓	NA	✓	NA	NA	NA	*	*	*	*	*	✓	3/7	42.9%
Massacand et al. 2009	C	✓	✓	✓	✓	✓	✓	*	*	*	*	*	✓	7/7	100%
Svensson et al. 2009	C	✓	✓	✓	✓	✓	✓	*	*	*	*	*	✓	7/7	100%
Hepworth et al. 2009	C	✓	✓	✓	✓	✓	✓	*	*	*	*	*	✓	7/7	100%
Hasnain et al. 2010	C	✓	✓	✓	✓	✓	✓	*	*	*	*	*	✓	7/7	100%
Angyalosi et al. 2001	S	✓	✓	✓	✓	NA	*	*	*	*	*	*	Y	4/5	80%
Byström et al. 2006	S	✓	✓	✓	✓	NA	*	*	*	*	*	*	NA	4/6	66.7%
Singh et al. 2006	S	✓	✓	✓	✓	NA	*	*	*	*	*	*	NA	4/6	66.7%
Burke et al. 2010	S	✓	✓	✓	NA	NA	*	*	*	*	*	*	NA	3/6	50%
de Oliveira et al. 2010	S	✓	✓	✓	Y	NA	*	*	*	*	*	*	✓	4/5	80%
Burke et al. 2011	S	✓	✓	✓	NA	NA	*	*	*	*	*	*	NA	3/6	50%
Perry et al. 2011	S	✓	✓	✓	NA	NA	*	*	*	*	*	*	NA	3/6	50%
Zhang et al. 2011	S	✓	✓	✓	✓	NA	*	*	*	*	*	*	NA	4/6	66.7%
Ray et al. 2012	S	✓	NA	✓	✓	NA	NA	*	*	*	*	*	✓	4/7	57.1%
de la Torre et al. 2012	S	✓	NA	✓	NA	NA	*	*	*	*	*	*	NA	2/6	33.3%
Ragno et al., 2001	TBC	✓	✓	*	*	*	*	*	*	✓	*	✓	NA	4/5	80%
Xu et al., 2003	TBC	✓	✓	*	*	*	*	*	*	✓	✓	✓	✓	6/6	100%
Keller et al., 2004	TBC	✓	✓	*	*	*	*	*	*	✓	✓	✓	✓	6/6	100%
Volpe et al., 2006	TBC	✓	✓	*	*	*	*	*	*	✓	*	NA	✓	4/5	80%
Orlova et al., 2006	TBC	✓	✓	*	*	*	*	*	*	NA	NA	NA	NA	2/6	33.3%
Silver et al., 2009	TBC	✓	✓	*	*	*	*	*	*	✓	✓	✓	✓	6/6	100%
Maddocks et al., 2009	TBC	✓	✓	*	*	*	*	*	*	✓	*	NA	NA	3/5	60%
Beisiegel et al., 2009	TBC	✓	✓	*	*	*	*	*	*	✓	✓	NA	✓	5/6	83.3%
Sharbati et al., 2011	TBC	✓	✓	*	*	*	*	*	*	✓	✓	✓	✓	6/6	100%
Magee et al., 2012	TBC	✓	✓	*	*	*	*	*	*	✓	✓	✓	✓	6/6	100%
Total		60/60	53/60	49/50	27/39	9/40	10/31	10/18	4/4	23/24	20/21	11/24	28/59		
%		100%	88.3%	98.0%	69.2%	22.5%	32.3%	55.6%	100%	95.8%	95.2%	45.8%	47.5%		

L = *Leishmania*, T = *Toxoplasma*, P = *Plasmodium*, C = colitis induced by *Trichuris*, S = *Schistosoma* and TBC = tuberculosis. Criteria: P1 (species), P2 (strain), P3 (stage), P4 (species and strain), P5 (age), P6 (gender), P7 (parasite collection sample); P8 (cell type), P9 (culture medium), P10 (supplements and antibiotics), P11 (temperature and CO_2 atmosphere), and P12 (time of growing of the parasite prior to infection).
✓: meets the criteria
NA: information not available
*: not applicable
