# Supplementary material for: The Quality of Methods Reporting in Parasitology Experiments
Source: PLoS One. 2014 Jul 30;9(7):e101131. doi: 10.1371/journal.pone.0101131 (PMC4116335; doi:10.1371/journal.pone.0101131)
Supplement: Table S6 — Quality measures of the studies that failed to supply any one of the criteria for minimal information about the host in Leishmania, Toxoplasma, Plasmodium, Trichuris, Schistosoma and Mycobacterium experiments. (PDF) [file pone.0101131.s006.pdf]

Table S6. Quality measures of the studies that failed to supply any one of the criteria for minimal information about the host in *Leishmania*, *Toxoplasma*, *Plasmodium*, *Trichuris*, *Schistosoma* and *Mycobacterium* experiments.

		Characteristics and culture conditions of the host models													
		Animal model					Cell model								
**Articles**	**Model**	**H1**	**H2**	**H3**	**H4**	**H5**	**H6**	**H7**	**H8**	**H9**	**H10**	**H11**	**H12**	**Total**	**%**
Park et al., 2000	L	✓	✓	✓	NA	NA	✓	✓	✓	NA	✓	NA	*	7/11	63.6%
Filippi et al., 2003	L	✓	✓	NA	NA	NA	*	*	*	*	*	*	*	2/5	40%
Bertholet et al., 2005	L	✓	NA	NA	NA	NA	*	*	*	*	*	*	*	1/5	20%
Kinjyo et al., 2006	L	✓	✓	NA	NA	NA	*	*	*	*	*	*	*	2/5	40%
Brunner et al., 2007	L	✓	✓	NA	NA	NA	*	*	*	*	*	*	*	2/5	40%
Guerfali et al., 2008	L	*	*	*	*	*	✓	✓	✓	✓	✓	✓	✓	7/7	100%
Jayakumar et al., 2008	L	*	*	*	*	*	✓	*	*	✓	✓	✓	NA	4/5	80%
Ehrchen at al., 2010	L	✓	✓	✓	NA	NA	*	*	*	*	*	*	*	3/5	60%
Biswas et al., 2011	L	✓	✓	✓	NA	NA	✓	✓	✓	✓	✓	✓	NA	9/12	75%
de Carvalho et al., 2011	L	✓	NA	NA	NA	NA	✓	✓	NA	✓	✓	✓	✓	7/12	58.3%
Desolme et al., 2000	T	✓	✓	✓	NA	NA	*	*	*	*	*	*	*	3/5	60%
Gail et al., 2001	T	*	*	*	*	*	✓	✓	✓	NA	*	*	NA	3/5	60%
Fux et al., 2003	T	✓	✓	NA	NA	✓	*	*	*	*	*	*	*	3/5	60%
Tato et al., 2003	T	✓	✓	NA	NA	NA	*	*	*	*	*	*	*	2/5	40%
Okomo et al, 2006	T	*	*	*	*	*	✓	✓	✓	✓	*	*	NA	4/5	80%
Knight et al., 2006	T	*	*	*	*	*	✓	✓	✓	NA	*	*	NA	3/5	60%
Watford et al., 2008	T	✓	NA	✓	NA	NA	*	*	*	*	*	*	*	2/5	40%
Ju et al., 2009	T	*	*	*	*	*	✓	✓	✓	NA	*	*	NA	3/5	60%
Fang et al., 2009	T	✓	✓	✓	NA	NA	*	*	*	*	*	*	*	3/5	60%
Zhuo et al., 2011	T	✓	NA	NA	NA	NA	*	*	*	*	*	*	*	1/5	20%
Ylostalo et al., 2005	P	✓	NA	NA	NA	*	*	*	*	*	*	*	*	1/4	25%
Lovergrove et al., 2006	P	✓	✓	✓	NA	NA	*	*	*	*	*	*	*	3/5	60%
Delahaye et al., 2007	P	✓	✓	✓	NA	*	*	*	*	*	*	*	*	3/4	75%
Carapau et al., 2007	P	✓	NA	NA	NA	NA	✓	✓	✓	NA	✓	✓	*	6/11	54.5%
Miu et al., 2008	P	✓	✓	✓	NA	✓	*	*	*	*	*	*	*	4/5	80%
Randall et al., 2008	P	✓	✓	✓	NA	✓	*	*	*	*	*	*	*	4/5	80%
Oakley et al., 2008	P	✓	✓	✓	NA	NA	*	*	*	*	*	*	*	3/5	60%
Albuquerque et al, 2009	P	✓	✓	✓	✓	NA	✓	✓	✓	✓	✓	✓	✓	11/12	91.7%
Delic et al., 2011	P	✓	NA	✓	NA	✓	*	*	*	*	*	*	*	3/5	60%
Rosanas et al., 2012	P	✓	✓	✓	✓	NA	*	*	*	*	*	*	*	4/5	80%
Betts et al., 2001	C	✓	✓	NA	✓	NA	*	*	*	*	*	*	*	3/5	60%
Humphreys et al., 2004	C	✓	✓	NA	✓	NA	*	*	*	*	*	*	*	3/5	60%
Cliffe et al.,2005	C	✓	✓	✓	✓	NA	*	*	*	*	*	*	*	4/5	80%
Dixon et al., 2006	C	✓	✓	✓	✓	✓	*	*	*	*	*	*	*	5/5	100%
Bickle et al., 2007	C	✓	✓	✓	✓	NA	*	*	*	*	*	*	*	4/5	80%
Villarino et al., 2008	C	✓	✓	✓	✓	NA	*	*	*	*	*	*	*	4/5	80%
Massacand et al. 2009	C	✓	NA	NA	✓	NA	*	*	*	*	*	*	*	2/5	40%
Svensson et al. 2009	C	✓	✓	NA	✓	NA	*	*	*	*	*	*	*	3/5	60%
Hepworth et al. 2009	C	✓	✓	✓	✓	NA	*	*	*	*	*	*	*	4/5	80%
Hasnain et al. 2010	C	✓	✓	✓	✓	NA	*	*	*	*	*	*	*	4/5	80%
Angyalosi et al. 2001	S	✓	✓	NA	✓	NA	*	*	*	*	*	*	*	3/5	60%
Byström et al. 2006	S	✓	NA	NA	NA	NA	*	*	*	*	*	*	*	1/5	20%
Singh et al. 2006	S	✓	✓	✓	✓	NA	*	*	*	*	*	*	*	4/5	80%
Burke et al. 2010	S	✓	✓	✓	✓	NA	*	*	*	*	*	*	*	4/5	80%
de Oliveira et al. 2010	S	✓	N	N	✓	NA	*	*	*	*	*	*	*	2/3	66.7%
Burke et al. 2011	S	✓	✓	✓	NA	NA	*	*	*	*	*	*	*	3/5	60%
Perry et al. 2011	S	✓	✓	✓	NA	NA	*	*	*	*	*	*	*	3/5	60%
Zhang et al. 2011	S	✓	✓	✓	Y	NA	*	*	*	*	*	*	*	3/4	75%
Ray et al. 2012	S	✓	✓	✓	NA	NA	*	*	*	*	*	*	*	3/5	60%
de la Torre et al. 2012	S	✓	NA	NA	NA	✓	*	*	*	*	*	*	*	2/5	40%
Ragno et al., 2001	TBC	*	*	*	*	*	✓	*	*	✓	✓	NA	NA	3/5	60%
Xu et al., 2003	TBC	*	*	*	*	*	✓	✓	NA	✓	✓	✓	NA	5/7	71.4%
Keller et al., 2004	TBC	✓	✓	✓	✓	NA	✓	✓	NA	✓	✓	NA	✓	9/12	75%
Volpe et al., 2006	TBC	✓	NA	NA	*	*	✓	✓	✓	✓	✓	✓	✓	8/10	80%
Orlova et al., 2006	TBC	*	*	*	*	*	✓	✓	NA	✓	✓	NA	NA	4/7	57.1%
Silver et al., 2009	TBC	✓	NA	NA	*	*	✓	✓	✓	✓	✓	*	✓	7/9	77.8%
Maddocks et al., 2009	TBC	✓	NA	NA	*	*	✓	✓	✓	✓	✓	NA	✓	7/10	70%
Beisiegel et al., 2009	TBC	✓	NA	NA	✓	NA	*	*	*	*	*	*	*	2/5	40%
Sharbati et al., 2011	TBC	✓	NA	NA	*	*	✓	✓	✓	✓	✓	✓	✓	8/10	80%
Magee et al., 2012	TBC	✓	✓	✓	✓	*	✓	✓	✓	✓	✓	✓	✓	11/11	100%
Total		51/51	35/50	28/50	19/46	6/44	20/20	18/18	14/18	15/20	16/16	10/15	9/18		
%		100%	70%	56%	41.3%	13.6%	100%	100%	77.8%	75%	100%	66.7%	50%		

L = *Leishmania*, T = *Toxoplasma*, P = *Plasmodium*, C = colitis induced by *Trichuris*, S = *Schistosoma* and TBC = tuberculosis. Criteria: H1 (species and strain), H2 (age); H3 (gender), H4 (light and dark cycle), H5 (method of sacrifice), H6 (cell type), H7 (culture medium), H8 (supplements and antibiotics), H9 (temperature and CO_2 atmosphere), H10 (organ or tissue which takes the primary culture), H11 (method of purification for establishing primary culture), and H12 (time of growing of the cell prior to infection).

✓: meets the criteria

NA: information not available

*: not applicable
