# Supplementary material for: The Quality of Methods Reporting in Parasitology Experiments
Source: PLoS One. 2014 Jul 30;9(7):e101131. doi: 10.1371/journal.pone.0101131 (PMC4116335; doi:10.1371/journal.pone.0101131)
Supplement: Table S7 — Quality measures of the studies that failed to supply any one of the criteria for minimal information about the experimental infection in Leishmania, Toxoplasma, Plasmodium, Trichuris, Schistosoma and Mycobacterium experiments. (PDF) [file pone.0101131.s007.pdf]

Table S7. Quality measures of the studies that failed to supply any one of the criteria for minimal information about the experimental infection in *Leishmania*, *Toxoplasma*, *Plasmodium*, *Trichuris*, *Schistosoma* and *Mycobacterium* experiments.

		Characteristics of the experiment													
		Animal models					Cellular models				Parasite				
**Articles**	**Model**	**I1**	**I2**	**I3**	**I4**	**I5**	**I6**	**I7**	**I8**	**I9**	**I10**	**I11**	**I12**	**Total**	**%**
Park et al., 2000	L	✓	✓	NA	✓	NA	NA	*	*	*	NA	✓	✓	5/9	55.6%
Filippi et al., 2003	L	✓	✓	NA	NA	NA	✓	*	*	*	NA	NA	✓	4/9	44.4%
Bertholet et al., 2005	L	✓	✓	NA	✓	NA	*	NA	✓	NA	✓	✓	✓	7/11	63.6%
Kinjyo et al., 2006	L	✓	✓	NA	✓	NA	*	*	*	*	NA	NA	✓	4/8	50%
Brunner et al., 2007	L	✓	✓	✓	✓	NA	*	*	*	*	NA	Y	✓	5/7	71.4%
Guerfali et al., 2008	L	*	*	*	*	*	✓	NA	✓	✓	NA	NA	✓	4/7	57.1%
Jayakumar et al., 2008	L	*	*	*	*	*	*	NA	✓	✓	✓	✓	✓	5/6	83.3%
Ehrchen at al., 2010	L	✓	✓	✓	NA	NA	*	*	*	*	NA	NA	✓	4/8	50%
Biswas et al., 2011	L	✓	✓	NA	NA	NA	NA	NA	*	*	NA	NA	✓	3/10	30%
de Carvalho et al., 2011	L	*	*	*	*	*	NA	NA	✓	✓	NA	✓	✓	4/7	57.1%
Desolme et al., 2000	T	✓	✓	NA	✓	✓	*	*	*	*	NA	✓	✓	6/8	75%
Gail et al., 2001	T	*	*	*	*	*	*	NA	✓	✓	NA	✓	✓	4/6	66.7%
Fux et al., 2003	T	✓	✓	✓	✓	✓	*	*	*	*	NA	✓	✓	7/8	87.5%
Tato et al., 2003	T	✓	✓	NA	✓	✓	*	*	*	*	NA	✓	✓	6/8	75%
Okomo et al, 2006	T	*	*	*	*	*	*	NA	✓	NA	NA	✓	✓	3/6	50%
Knight et al., 2006	T	*	*	*	*	*	*	NA	✓	NA	NA	✓	✓	3/6	50%
Watford et al., 2008	T	✓	✓	NA	✓	✓	*	*	*	*	NA	✓	✓	6/8	75%
Ju et al., 2009	T	*	*	*	*	*	*	NA	✓	NA	NA	✓	✓	3/6	50%
Fang et al., 2009	T	✓	✓	✓	NA	✓	*	*	*	*	NA	✓	✓	6/8	75%
Zhuo et al., 2011	T	✓	NA	NA	NA	NA	*	*	*	*	NA	✓	✓	3/8	37.5%
Ylostalo et al., 2005	P	✓	✓	✓	✓	NA	*	*	*	*	NA	✓	✓	6/8	75%
Lovergrove et al., 2006	P	✓	✓	✓	NA	NA	*	*	*	*	NA	✓	✓	5/8	62.5%
Delahaye et al., 2007	P	✓	✓	✓	✓	✓	*	*	*	*	NA	✓	✓	7/8	87.5%
Carapau et al., 2007	P	✓	✓	✓	✓	NA	✓	NA	✓	NA	NA	✓	✓	8/12	66.7%
Miu et al., 2008	P	✓	✓	✓	NA	NA	*	*	*	*	NA	NA	✓	4/8	50%
Randall et al., 2008	P	✓	NA	✓	✓	✓	*	*	*	*	NA	✓	✓	6/8	75%
Oakley et al., 2008	P	✓	✓	NA	✓	NA	*	*	*	*	NA	NA	NA	3/8	37.5%
Albuquerque et al, 2009	P	✓	✓	NA	NA	NA	NA	NA	✓	NA	NA	✓	✓	5/12	41.7%
Delic et al., 2011	P	✓	✓	✓	✓	✓	*	*	*	*	NA	✓	✓	7/8	87.5%
Rosanas et al., 2012	P	✓	✓	✓	✓	✓	*	*	*	*	NA	✓	✓	7/8	87.5%
Betts et al., 2001	C	✓	✓	NA	✓	*	*	*	*	*	✓	*	✓	5/6	83.3%
Humphreys et al., 2004	C	✓	✓	NA	✓	*	*	*	*	*	✓	*	✓	5/6	83.3%
Cliffe et al.,2005	C	✓	✓	✓	✓	*	*	*	*	*	✓	*	✓	6/6	100%
Dixon et al., 2006	C	✓	✓	✓	✓	*	*	*	*	*	✓	*	✓	6/6	100%
Bickle et al., 2007	C	✓	✓	✓	✓	*	*	*	*	*	✓	*	✓	6/6	100%
Villarino et al., 2008	C	✓	✓	NA	✓	*	*	*	*	*	NA	*	✓	4/6	66.7%
Massacand et al. 2009	C	✓	✓	✓	✓	*	*	*	*	*	✓	*	✓	6/6	100%
Svensson et al. 2009	C	✓	✓	✓	✓	*	*	*	*	*	✓	*	✓	6/6	100%
Hepworth et al. 2009	C	✓	✓	✓	✓	*	*	*	*	*	✓	*	✓	6/6	100%
Hasnain et al. 2010	C	✓	✓	✓	✓	*	*	*	*	*	✓	*	✓	6/6	100%
Angyalosi et al. 2001	S	✓	✓	NA	✓	✓	*	*	*	*	NA	✓	✓	6/8	75%
Byström et al. 2006	S	✓	✓	NA	NA	NA	*	*	*	*	NA	✓	✓	4/8	50%
Singh et al. 2006	S	✓	✓	NA	NA	NA	*	*	*	*	NA	✓	✓	4/8	50%
Burke et al. 2010	S	✓	✓	✓	Y	NA	*	*	*	*	NA	✓	✓	5/7	71.4%
de Oliveira et al. 2010	S	✓	✓	✓	NA	NA	*	*	*	*	NA	✓	✓	5/8	62.5%
Burke et al. 2011	S	✓	✓	NA	✓	NA	*	*	*	*	NA	✓	✓	5/8	62.5%
Perry et al. 2011	S	✓	✓	NA	✓	NA	*	*	*	*	NA	✓	✓	5/8	62.5%
Zhang et al. 2011	S	✓	✓	✓	✓	NA	*	*	*	*	NA	✓	✓	6/8	75%
Ray et al. 2012	S	✓	✓	Y	NA	NA	*	*	*	*	NA	✓	✓	4/7	57.1%
de la Torre et al. 2012	S	✓	✓	NA	NA	NA	*	*	*	*	NA	✓	✓	4/8	50%
Ragno et al., 2001	TBC	*	*	*	*	*	*	NA	✓	NA	✓	*	✓	3/5	60%
Xu et al., 2003	TBC	*	*	*	*	*	*	NA	✓	✓	NA	*	✓	3/5	60%
Keller et al., 2004	TBC	*	*	*	*	*	NA	✓	✓	✓	✓	*	✓	5/6	83.3%
Volpe et al., 2006	TBC	*	*	*	*	*	✓	NA	✓	✓	✓	*	✓	5/6	83.3%
Orlova et al., 2006	TBC	*	*	*	*	*	NA	NA	✓	✓	NA	*	✓	3/6	50%
Silver et al., 2009	TBC	*	*	*	*	*	✓	NA	✓	✓	NA	*	✓	4/6	66.7%
Maddocks et al., 2009	TBC	*	*	*	*	*	NA	NA	✓	NA	NA	*	✓	2/6	33.3%
Beisiegel et al., 2009	TBC	✓	✓	✓	✓	NA	*	*	*	*	✓	*	✓	6/7	85.7%
Sharbati et al., 2011	TBC	*	*	*	*	*	✓	NA	✓	NA	✓	*	✓	4/6	66.7%
Magee et al., 2012	TBC	*	*	*	*	*	✓	NA	✓	NA	✓	*	✓	4/6	66.7%
Total		44/44	42/44	23/43	30/43	10/34	7/14	1/20	19/19	9/19	17/60	32/39	59/60		
%		100%	95.5%	53.5%	69.8%	29.4%	50%	5%	100%	47.4%	28.3%	82.1%	98.3%		

L = *Leishmania*, T = *Toxoplasma*, P = *Plasmodium*, C = colitis induced by *Trichuris*, S = *Schistosoma* and TBC = tuberculosis. Criteria: I1 (inoculum –parasite per animal), I2 (way of inoculation), I3 (medium of inoculation), I4 (parasitaemia and post infection time in which parasitaemia was measured), I5 (mortality of animals post infection), I6 (purity of primary culture), I7 (viability of the cells prior infection), I8 (ratio –parasites per cell), I9 (percentage infected cells), I10 (viability of the parasite prior infection), I11 (purity of the infective form of the parasite), and I12 (duration of infection).

✓: meets the criteria

NA: information not available

*****: not applicable
